# Supplementary material for: Silica-copper catalyst interfaces enable carbon-carbon coupling towards ethylene electrosynthesis
Source: Nat Commun. 2021 May 14;12:2808. doi: 10.1038/s41467-021-23023-0 (PMC8121866; doi:10.1038/s41467-021-23023-0)
Supplement: Supplementary file 1 — Supplementary Information [file 41467_2021_23023_MOESM1_ESM.pdf]

Supplementary Information for

Silica-copper catalyst interfaces enable carbon-carbon coupling towards ethylene electrosynthesis

Jun Li†, Adnan Ozden†, Mingyu Wan†, Yongfeng Hu, Fengwang Li, Yuhang Wang, Reza R. Zamani, Dan Ren, Ziyun Wang, Yi Xu, Dae-Hyun Nam, Joshua Wicks, Bin Chen, Xue Wang, Mingchuan Luo, Michael Graetzel, Fanglin Che, Edward H. Sargent* and David Sinton**

†Equally contributed authors: Jun Li, Adnan Ozden and Mingyu Wan.

*Correspondence: fanglin_che@uml.edu; ted.sargent@utoronto.ca; sinton@mie.utoronto.ca

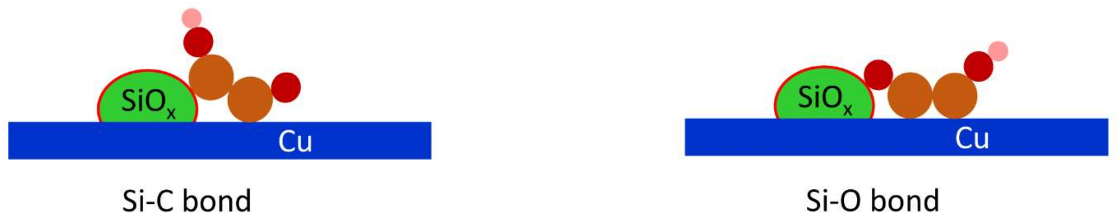

Supplementary Fig. 1 A schematic view of the Cu-SiO$_x$ interface step sites for enhancing the OCCOH adsorption via forming Si-C (left) and Si-O (right) bonds. Color-coded atoms represent C (brown), O (red) and H (pink).

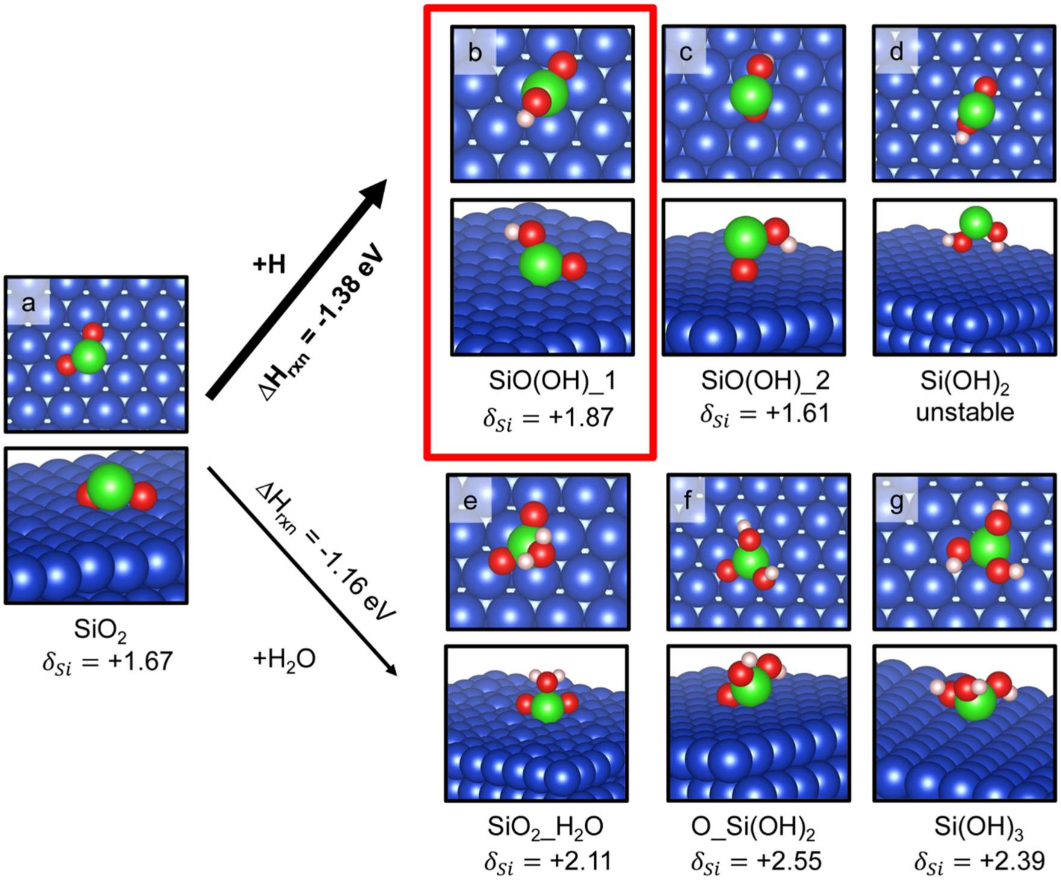

Supplementary Fig. 2 The energetics of forming possible geometries of the Cu-SiO_x catalyst at a silica coverage of 1/16 monolayer (1.6% silica loading) and the corresponding Si oxidation state (δ_{Si}). **a** SiO_2 adsorption geometry. **b** Energetically favorable SiO(OH) formation from SiO_2 via hydrogen proton transfer under hydrogen-proton-riched environment. **c** Another possible adsorption geometry of SiO(OH) but energetically much less favorable compared to the geometry of **b**. **d** The formation of unstable $Si(OH)_2$ over the Cu surface after additional hydrogen proton transfer to SiO(OH) in **b**. **e** The formation of SiO_2_H_2O from SiO_2 via adsorbed water under water-enriched environment, which is energetically less favourable than the hydrogen proton transfer path in **b**. **f** The formation of O_$Si(OH)_2$ from the water dissociation of SiO_2_H_2O in **e**. **g** The formation of $Si(OH)_3$ from hydrogen proton transfer to O_$Si(OH)_2$ in **f**. The energetics of each geometry are shown in **Supplementary Table 1**. Color-coded atoms represent Cu (blue), Si (green), O (red) and H (pink).

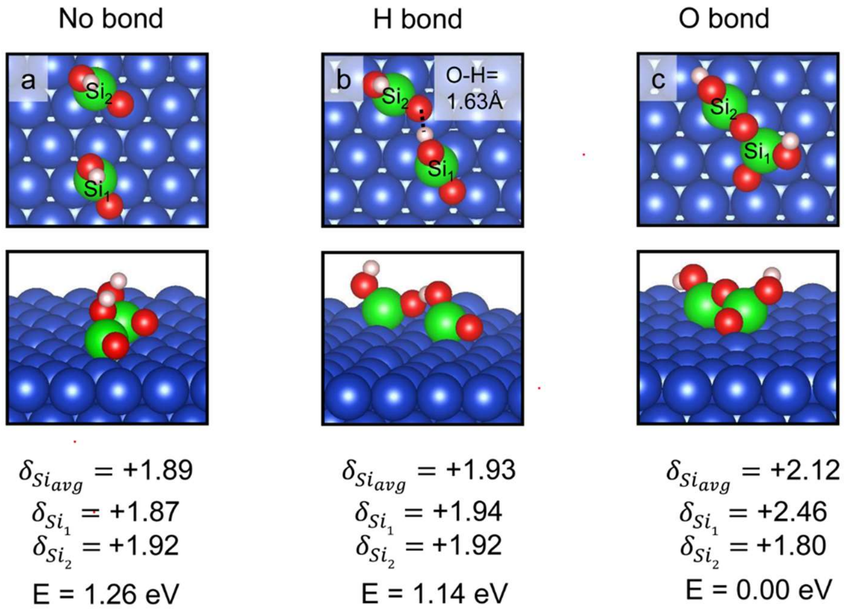

Supplementary Fig. 3 The energetics of the possible geometries of the Cu-SiO_x catalyst at a silica coverage of 1/8 monolayer (3.1% silica loading) and the corresponding Si oxidation state (δ_{Si}). a The geometry with no bond formation between two SiO_2 molecules over the Cu surface. **b** The formation of hydrogen bond (O-H bond length of 1.63 Å) between two SiO_2 molecules over the Cu surface. **c** The formation of oxygen bond between two SiO_2 molecules over the Cu surface. The energies (*E*) reported here are all referred to the most favourable configuration energy in **c**. Color-code refers to Supplementary Fig. 2.

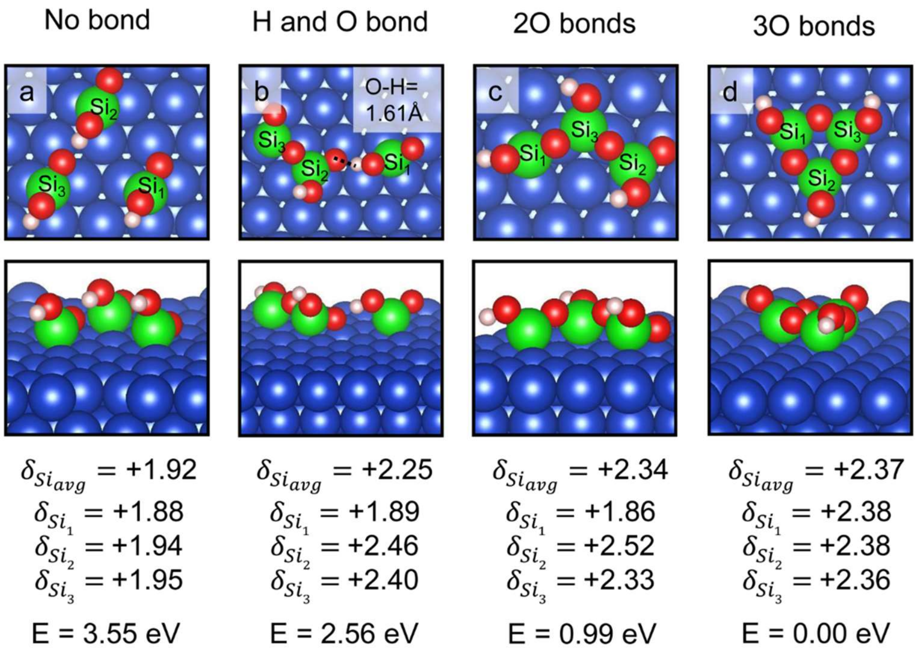

Supplementary Fig. 4 The energetics of the possible geometries of the Cu-SiO_x catalyst at a silica coverage of 3/16 monolayer (4.7% silica loading) and the corresponding Si oxidation state (δ_{Si}). a The geometry with no bond formation among three SiO_2 molecules over the Cu surface. **b** The formation of one hydrogen bond (O-H bond length of 1.61 Å) and one oxygen bond among three SiO_2 molecules over the Cu surface. **c** The formation of two oxygen bonds among three SiO_2 molecules over the Cu surface. **d** The formation of three oxygen bonds among three SiO_2 molecules over the Cu surface. The energies (*E*) reported here are all referred to the most favourable configuration energy in **d**. Color-code refers to Supplementary Fig. 2.

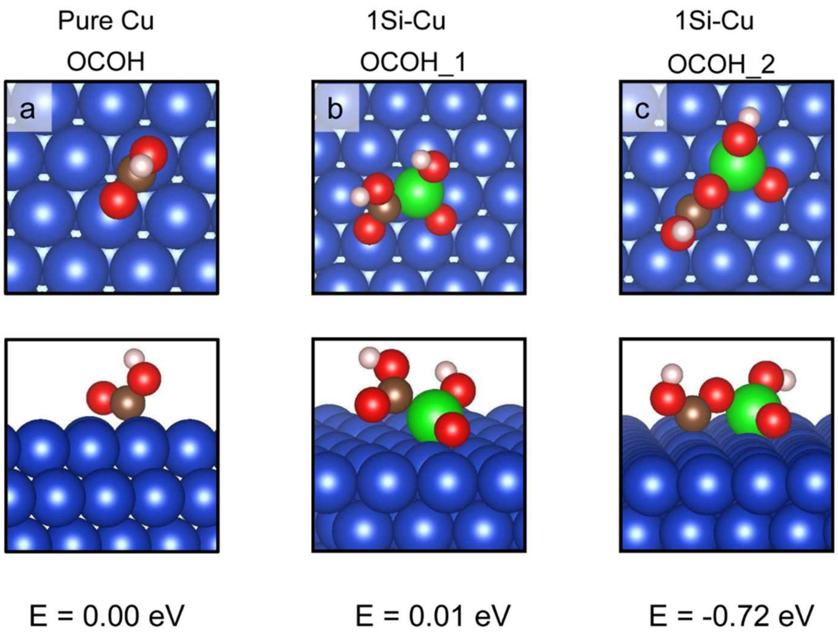

Supplementary Fig. 5 The adsorption of key intermediate OCOH over the Cu(111) and Cu-SiO$_x$ catalysts with a silica surface coverage of 1/16 ML (1.6% silica loading). a OCOH adsorption over the pure Cu catalyst. **b, c** OCOH adsorption over the Cu-SiO$_x$ catalyst with the formation of Si-C bond in **b** and Si-O bond in **c**. The energy (*E*) reported here is the adsorption energy difference compared to the configuration energy in **a**. Color-code refers to Supplementary Fig. 2.

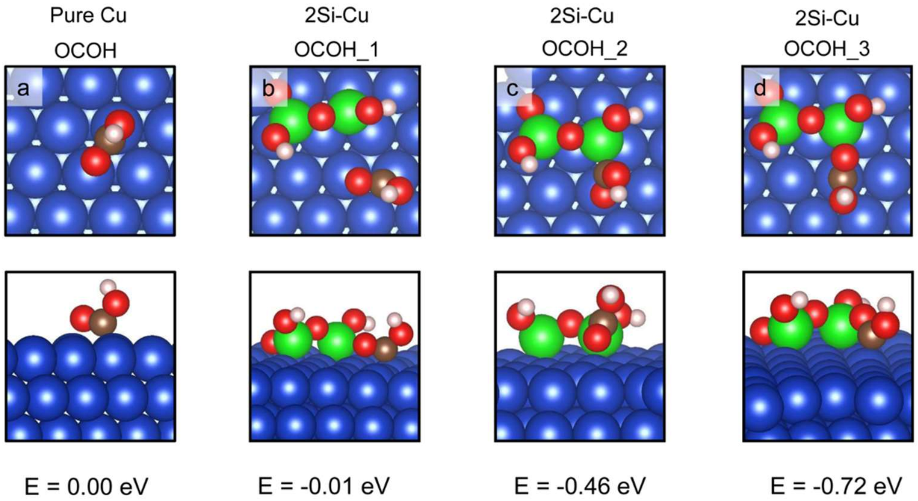

Supplementary Fig. 6 The adsorption of key intermediate OCOH over the Cu(111) and Cu-SiO$_x$ catalysts with a silica surface coverage of 1/8 ML (3.1% silica loading). a The OCOH adsorption over the pure Cu catalyst. **b-d** The OCOH adsorption over the Cu-SiO$_x$ catalyst with no bond in **b**, Si-C bond in **c** and Si-O bond in **d** formations between OCOH and silica. The energy (*E*) reported here is the adsorption energy difference compared to the configuration energy in **a**. Color-code refers to Supplementary Fig. 2.

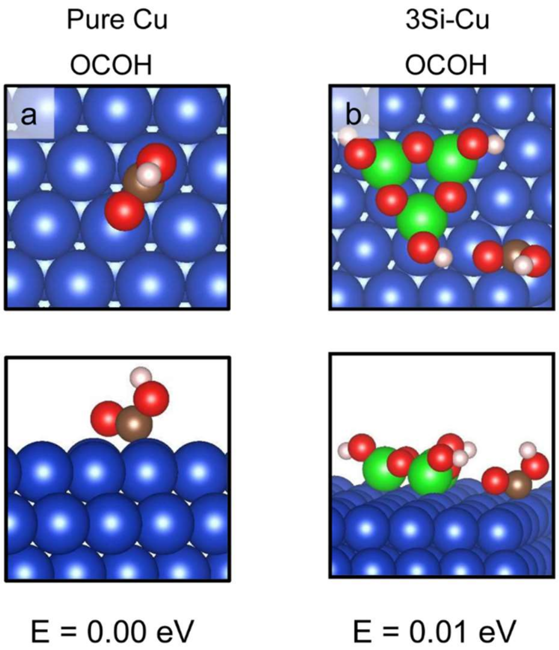

Supplementary Fig. 7 The adsorption of key intermediate OCOH over the Cu(111) and Cu-SiO$_x$ catalysts with a silica surface coverage of 3/16 ML (4.7% silica loading). a, b The OCOH adsorption over the pure Cu catalyst in **a** and the Cu-SiO$_x$ catalyst with no bond formation between OCOH and silica in **b**. The energy (*E*) reported here is the adsorption energy difference compared to the configuration energy in **a**. Color-code refers to Supplementary Fig. 2.

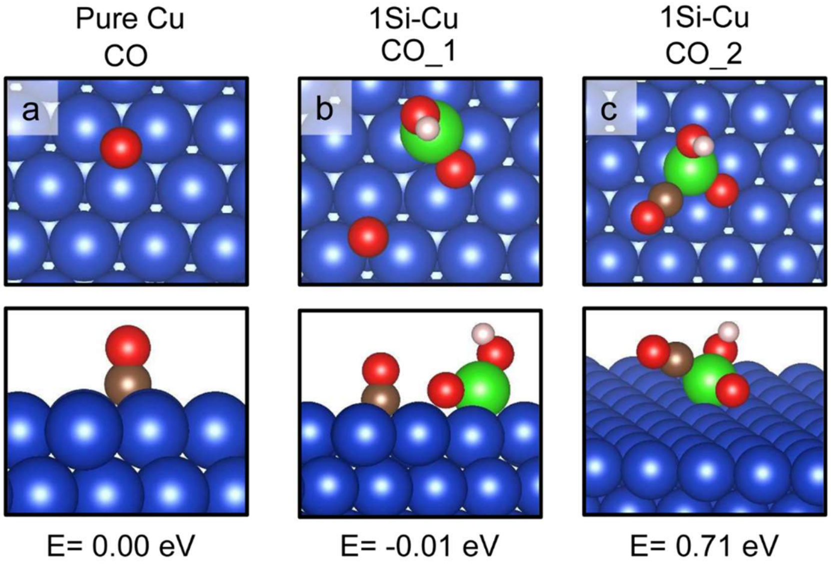

Supplementary Fig. 8 The adsorption of key intermediate CO over the Cu(111) and Cu-SiO_x catalysts with a silica surface coverage of 1/16 ML (1.6% silica loading). a, b The CO adsorption over the pure Cu catalyst in **a** and the Cu-SiO_x catalyst with no bond formation between CO and silica in **b**. **c** The Cu-SiO_x catalyst with Si-C bond formation between CO and silica. The energy (*E*) reported here is the adsorption energy difference compared to the configuration energy in **a**. Color-code refers to Supplementary Fig. 2.

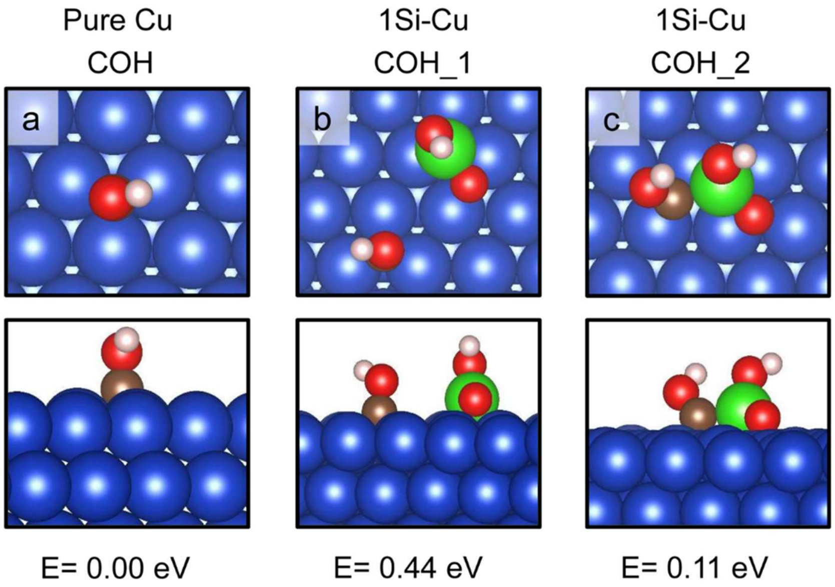

Supplementary Fig. 9 The adsorption of key intermediate COH over the Cu(111) and Cu-SiO_x catalysts with a silica surface coverage of 1/16 ML (1.6% silica loading). a The COH adsorption over the pure Cu catalyst. **b, c** The COH adsorption over the Cu-SiO_x catalyst with no bond in **b** and Si-C bond in **c** formations between COH and silica. The energy (*E*) reported here is the adsorption energy difference compared to the configuration energy in **a**. Color-code refers to Supplementary Fig. 2.

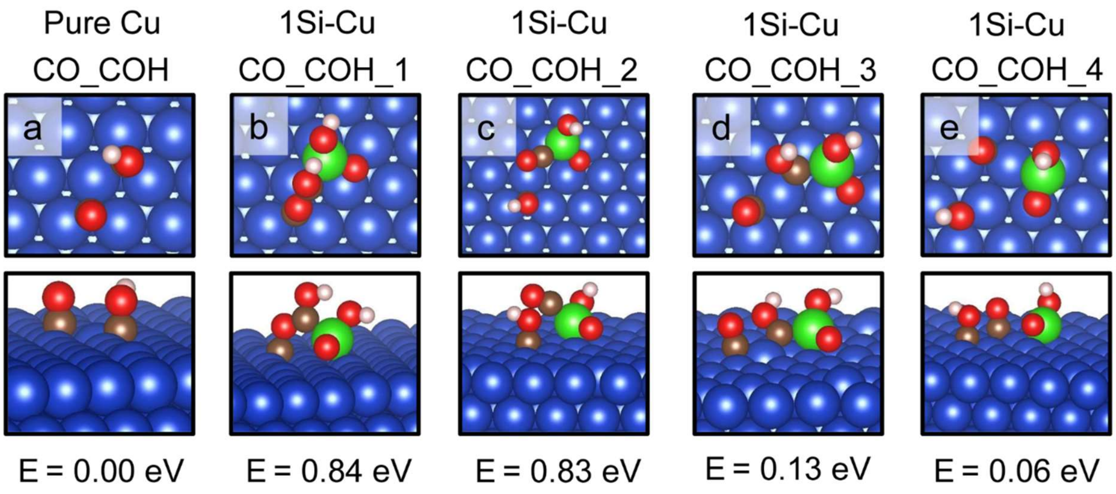

Supplementary Fig. 10 The coadsorption of key intermediates CO and COH over the Cu(111) and Cu-SiO$_x$ catalysts with a silica surface coverage of 1/16 ML (1.6% silica loading). a-c The CO_COH coadsorption over the pure Cu catalyst in **a** and the Cu-SiO$_x$ catalyst with COH in **b** and CO in **c** adsorption on silica. **d** The CO_COH coadsorption over the Cu-SiO$_x$ catalyst with COH binding to Cu and silica. **e** The CO_COH coadsorption over the Cu-SiO$_x$ catalyst with no bond formation among CO, COH and silica. The energy (*E*) reported here is the coadsorption energy difference compared to configuration energy in **a**. Color-code refers to Supplementary Fig. 2.

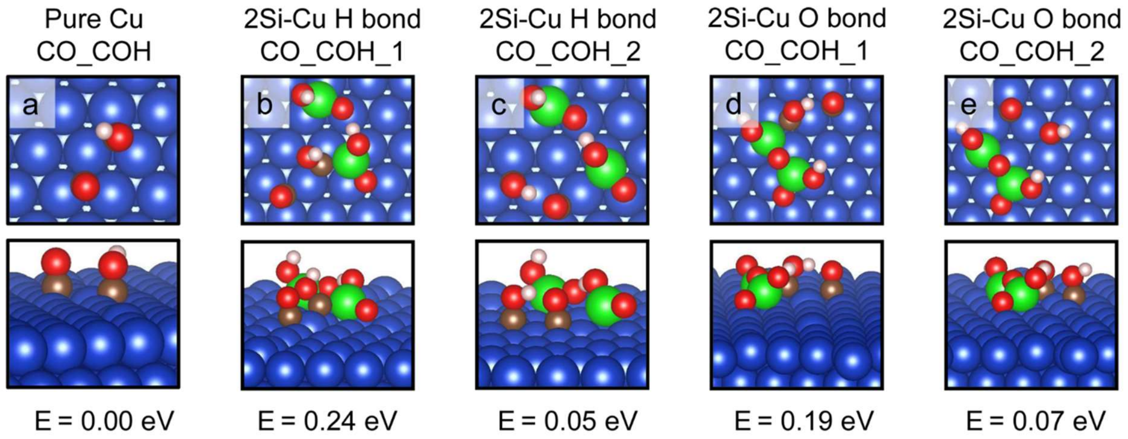

Supplementary Fig. 11 The coadsorption of key intermediates CO and COH over the Cu(111) and Cu-SiO$_x$ catalysts with a silica surface coverage of 1/8 ML (3.1% silica loading). a-c The CO_COH coadsorption over the pure Cu catalyst (**a**) and the Cu-SiO$_x$ catalyst (with H bond formation between two silica molecules) via Si-C bond (**b**) and no bond (**c**) formations between COH and silica. **d, e** The CO_COH coadsorption over the Cu-SiO$_x$ catalyst (with O bond formation between two silica molecules) via Si-C bond (**d**) and no-bond (**e**) formations between COH and silica. The energy (*E*) reported here is the co-adsorption energy difference compared to the configuration energy in (**a**). Color-code refers to Supplementary Fig. 2.

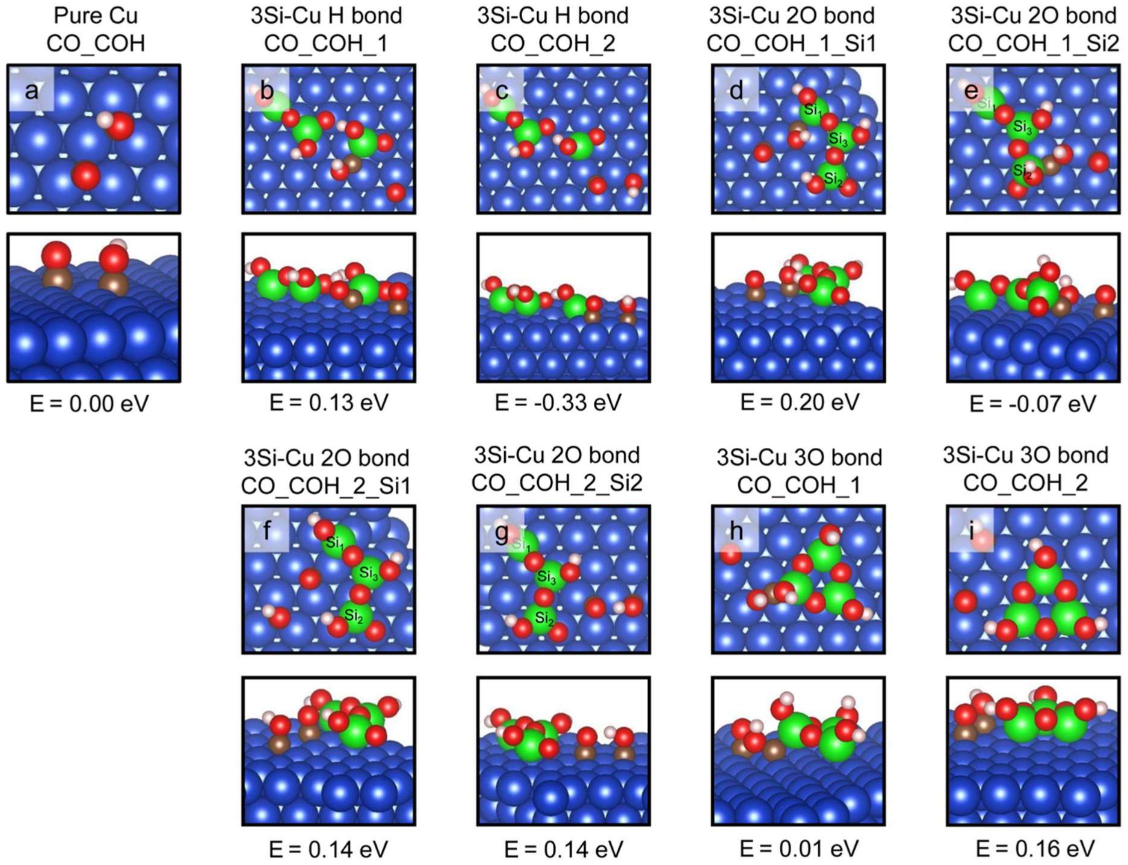

Supplementary Fig. 12 The coadsorption of key intermediates CO and COH over the Cu(111) and Cu-SiO_x catalysts with a silica surface coverage of 3/16 ML (4.7% silica loading). a-c The CO_COH coadsorption over the pure Cu catalyst (**a**) and the Cu-SiO_x catalyst (with one H and O bond formations among three silica molecules) via Si-C bond (**b**) and no-bond (**c**) formations between COH and silica. **d-g** The CO_COH coadsorption over the Cu-SiO_x catalyst (with two O bonds formations among three silica molecules) via Si_1-C bond (**d**), Si_2-C bond (**e**), and no-bond (**f, g**) formations between COH and silica. **h, i** The CO_COH coadsorption over the Cu-SiO_x catalyst (with three O bonds formations among three silica molecules) via Si-C bond (**h**) and no bond (**i**) formations between COH and silica. The energy (E) reported here is the co-adsorption energy difference compared to the configuration energy in (**a**). Color-code refers to Supplementary Fig. 2.

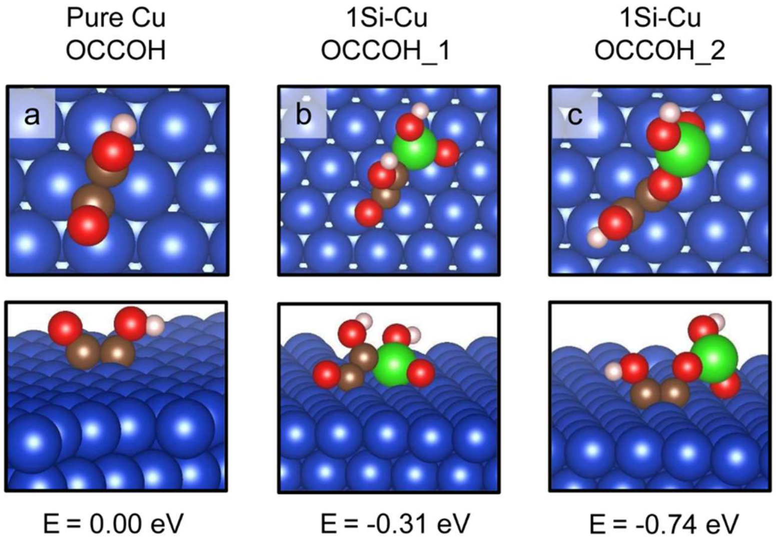

Supplementary Fig. 13 The adsorption of key intermediate OCCOH over the Cu(111) and Cu-SiO_x catalysts with a silica surface coverage of 1/16 ML (1.6% silica loading). a-c The OCCOH adsorption over the pure Cu catalyst (**a**) and the Cu-SiO_x catalyst with Si-C bond (**b**) and Si-O bond (**c**) formations between OCCOH and silica. The energy (*E*) reported here is the adsorption energy difference compared to the configuration energy in (**a**). Color-code refers to Supplementary Fig. 2.

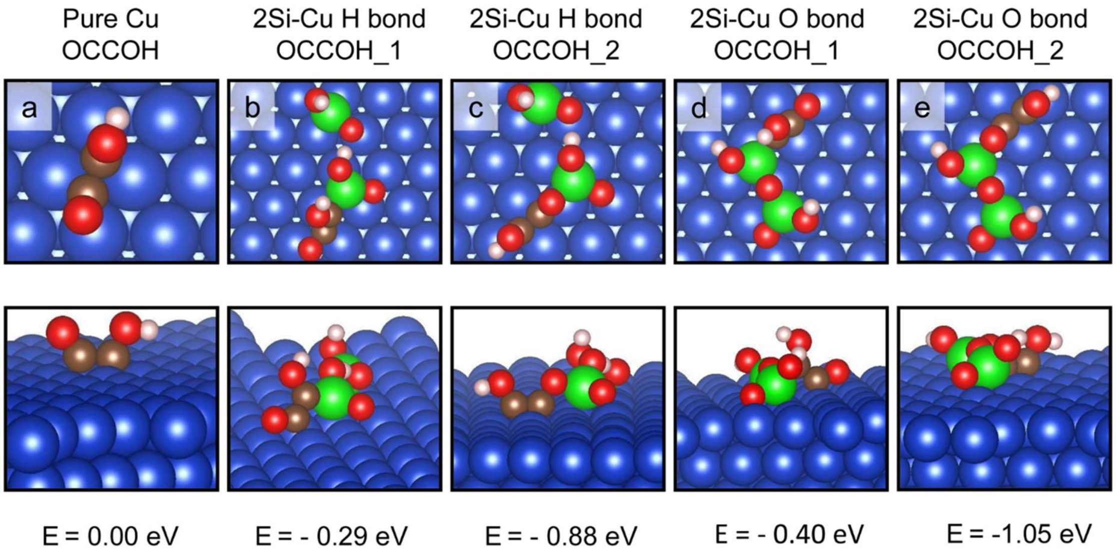

Supplementary Fig. 14 The adsorption of key intermediate OCCOH over the Cu(111) and Cu-SiO$_x$ catalysts with a silica surface coverage of 1/8 ML (3.1% silica loading). A-c The OCCOH adsorption over the pure Cu catalyst (**a**) and the Cu-SiO$_x$ catalyst (with H bond formation between two silica molecules) via Si-C bond (**b**) and Si-O bond (**c**) formations between OCCOH and silica. **D, e** The OCCOH adsorption over the Cu-SiO$_x$ catalyst (with O bond formation between two silica molecules) via Si-C bond (**d**) and Si-O bond € formations between OCCOH and silica. The energy (*E*) reported here is the adsorption energy difference compared to the configuration energy in (**a**). Color-code refers to Supplementary Fig. 2.

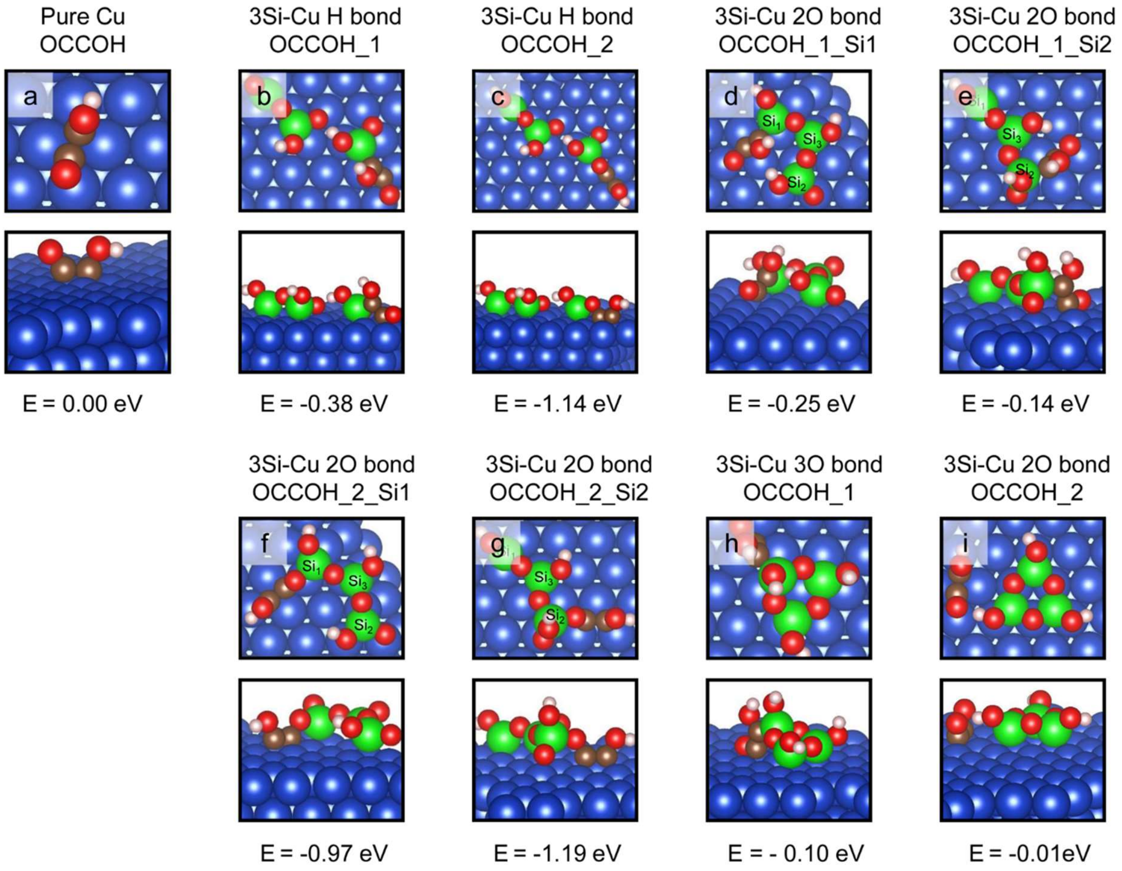

Supplementary Fig. 15 The adsorption of key intermediate OCCOH over the Cu(111) and Cu-SiO$_x$ catalysts with a silica surface coverage of 3/16 ML (4.7% silica loading). a-c The OCCOH adsorption over the pure Cu catalyst (**a**) and the Cu-SiO$_x$ catalyst (with one H and O bond formations among three silica molecules) via Si-C bond (**b**) and Si-O bond (**c**) formations between OCCOH and silica. **d-g** The OCCOH adsorption over the Cu-SiO$_x$ catalyst (with two O bonds formations among three silica molecules) via Si$_1$-C bond (**d**), Si$_2$-C bond (**e**), Si$_1$-O bond (**f**), and Si$_2$-O bond (**g**) formations between OCCOH and silica. **h, i** The OCCOH adsorption over the Cu-SiO$_x$ catalyst (with two O bonds formations among three silica molecules) via Si-C bond (**h**) and no bond (**i**) formations between OCCOH and silica. The energy (E) reported here is the adsorption energy difference compared to the configuration energy in (**a**). Color-code refers to Supplementary Fig. 2.

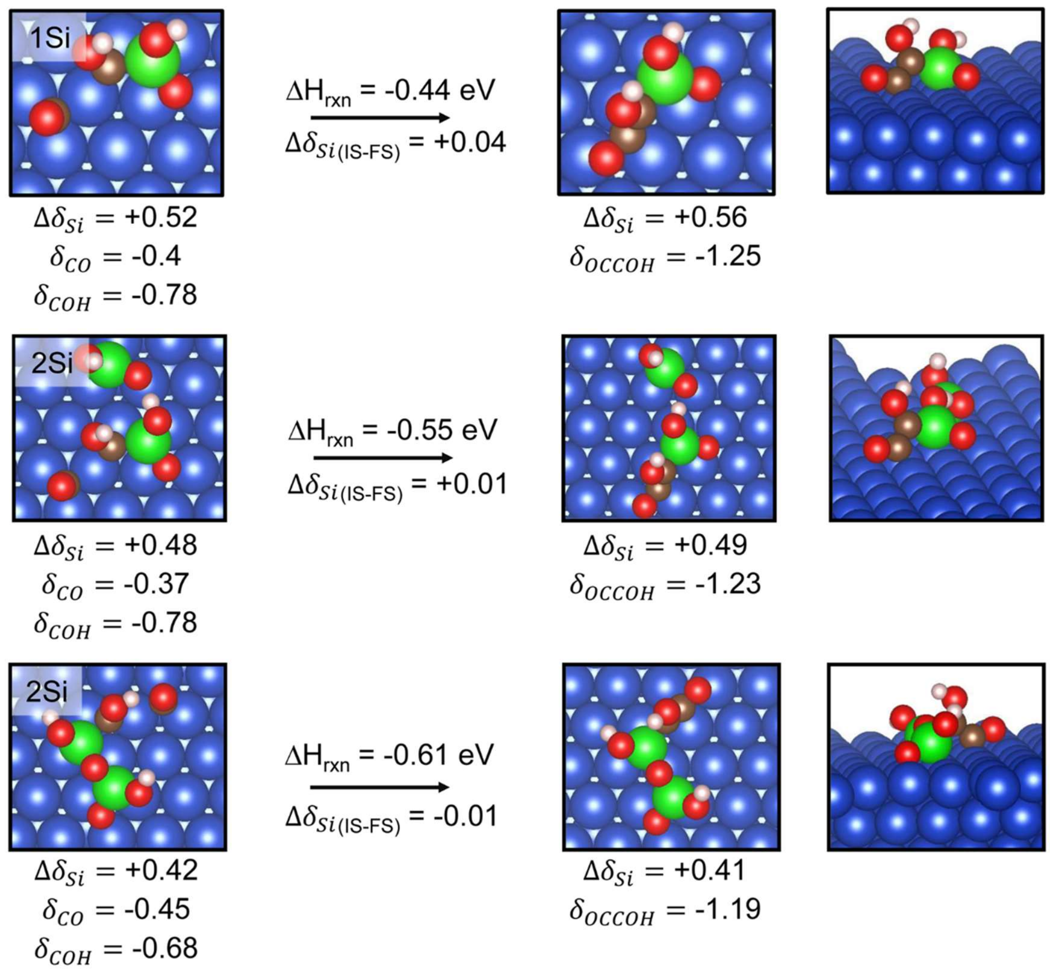

Supplementary Fig. 16 The role of Si-C bond (at a silica surface loading of 1/16 ML and 2/16 ML) in affecting the formation energy of OCCOH from co-adsorbed CO and COH. With the addition of silica, the Cu-SiO_x catalyst can generate a stable step surface configuration. Such a step surface configuration can lower the formation energy of OCCOH compared to the bare Cu catalyst (-0.02 eV). Bader charge analysis of $\Delta\delta_{Si}$ (the oxidation state difference of Si (δ_{Si}) in the presence and absence of adsorbates) represents the number of electron transfer between the adsorbates and silica. $\Delta\delta_{Si(\text{IS-FS})}$ shows the change in oxidation state of Si between the initial and final states. δ_{CO}, δ_{COH}, and δ_{OCCOH} represent the adsorbate CO, COH and OCCOH charge transfer with the surroundings. Color-code refers to Supplementary Fig. 2.

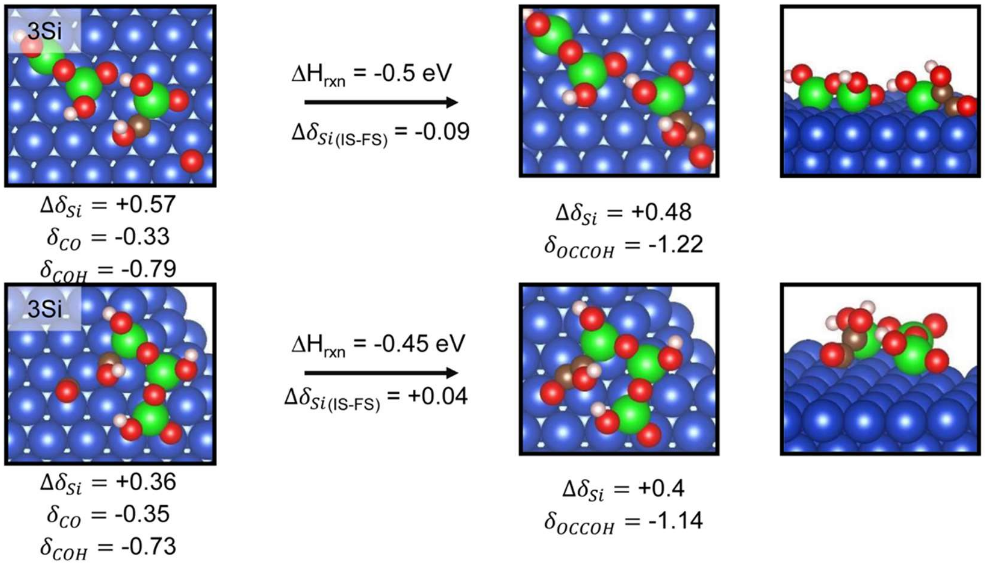

Supplementary Fig. 17 The role of Si-C bond (at a silica surface loading of 3/16 ML) in affecting the formation energy of OCCOH from co-adsorbed CO and COH. With the addition of silica, the Cu-SiO_x catalyst can generate a stable step surface configuration. Such a step surface configuration can lower the formation energy of OCCOH compared the bare Cu catalyst (-0.02 eV). Bader charge analysis of $\Delta\delta_{Si}$ (the oxidation state difference of Si (δ_{Si}) in the presence and absence of adsorbates) represents the number of electron transfer between the adsorbates and silica. $\Delta\delta_{Si(\text{IS-FS})}$ shows the change in oxidation state of Si between the initial and final states. δ_{CO}, δ_{COH}, and δ_{OCCOH} represent the adsorbate CO, COH and OCCOH charge transfer with the surroundings. Color-code refers to Supplementary Fig. 2.

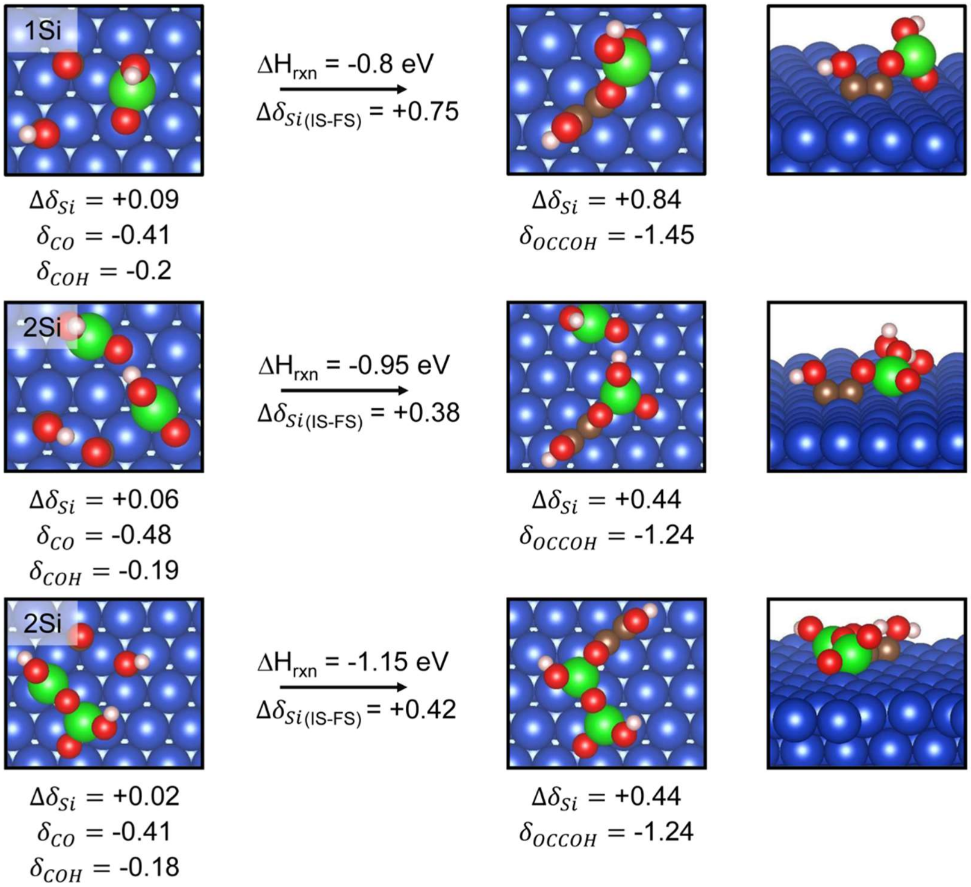

Supplementary Fig. 18 The role of Si-O bond (at a silica surface loading of 1/16 ML and 2/16 ML) in affecting the formation energy of $OCCOH^*$ from co-adsorbed CO and COH is that with the addition of silica, the Cu-SiO_x catalyst can generate a strong Si-O bond between OCCOH and silica. As a result, the Cu-SiO_x catalyst can lower the formation energy of OCCOH compared to the bare Cu catalyst (-0.02 eV). Bader charge analysis of $\Delta\delta_{Si}$ (the oxidation state difference of Si (δ_{Si}) in the presence and absence of adsorbates) represents the number of electron transfer between the adsorbates and silica. $\Delta\delta_{Si(\text{IS-FS})}$ shows the change in oxidation state of Si between the initial and final states. δ_{CO}, δ_{COH}, and δ_{OCCOH} represent the adsorbate CO, COH and OCCOH charge transfer with the surroundings. Color-code refers to Supplementary Fig. 2.

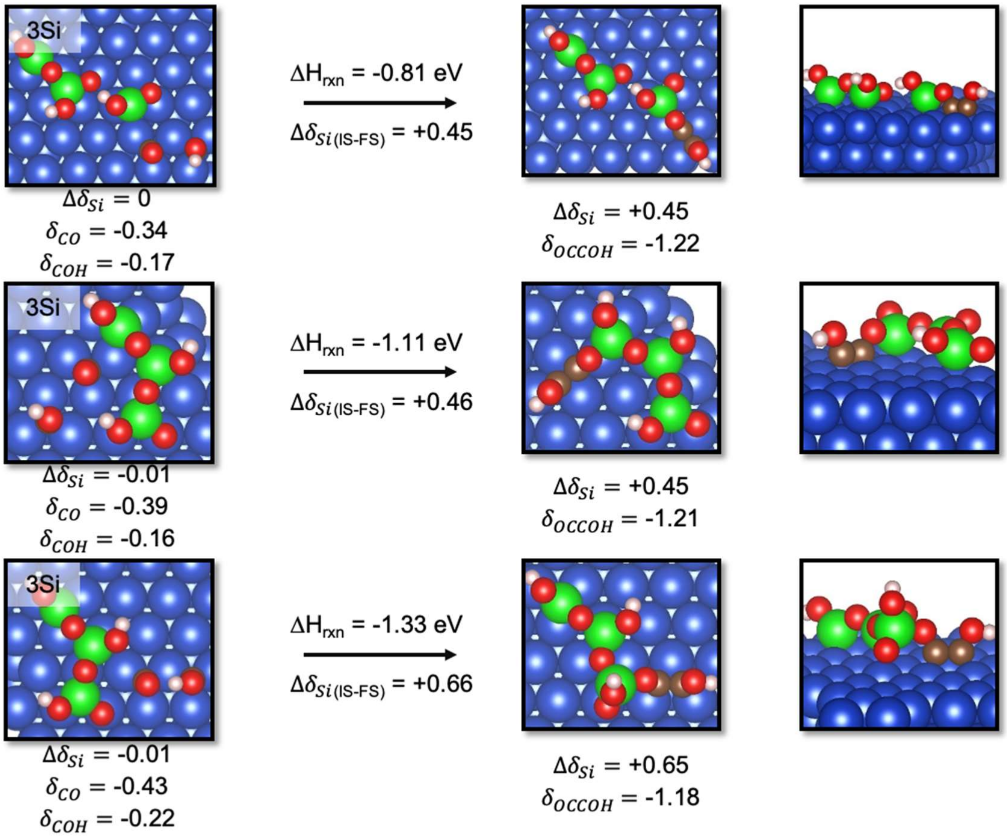

Supplementary Fig. 19 The role of Si-O bond (at a silica surface loading of 3/16 ML) in affecting the formation energy of OCCOH from co-adsorbed CO and COH is that with the addition of silica, the Cu-SiO_x catalyst can generate a strong Si-O bond between OCCOH and silica. As a result, the Cu-SiO_x catalyst can lower the formation energy of OCCOH compared to the bare Cu catalyst (-0.02 eV). Bader charge analysis of $\Delta\delta_{Si}$ (the oxidation state difference of Si (δ_{Si}) in the presence and absence of adsorbates) represents the number of electron transfer between the adsorbates and silica. $\Delta\delta_{Si(\text{IS-FS})}$ shows the change in oxidation state of Si between the initial and final states. δ_{CO}, δ_{COH}, and δ_{OCCOH} represent the adsorbate CO, COH and OCCOH charge transfer with the surroundings. Color-code refers to Supplementary Fig. 2.

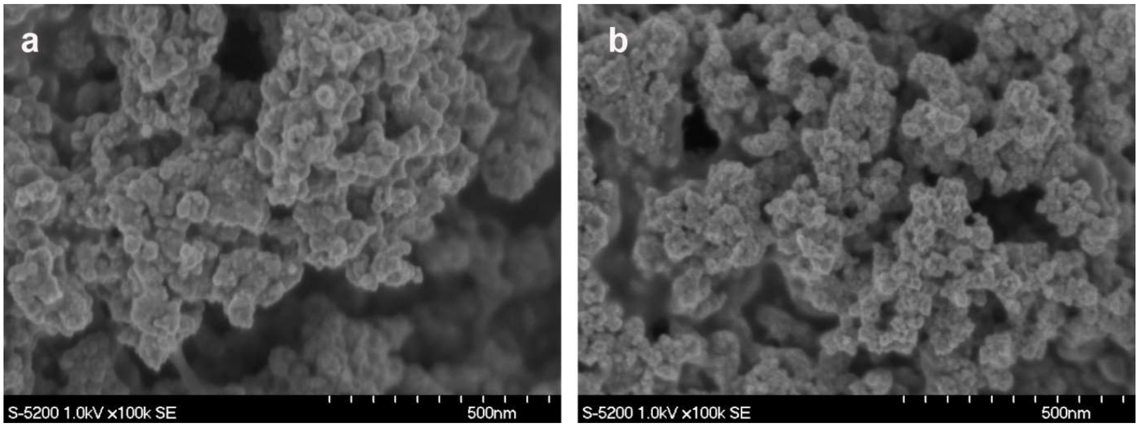

Supplementary Fig. 20 Material characterizations. a, b SEM images for the bare Cu (**a**) and Cu-SiO_x (**b**) catalysts.

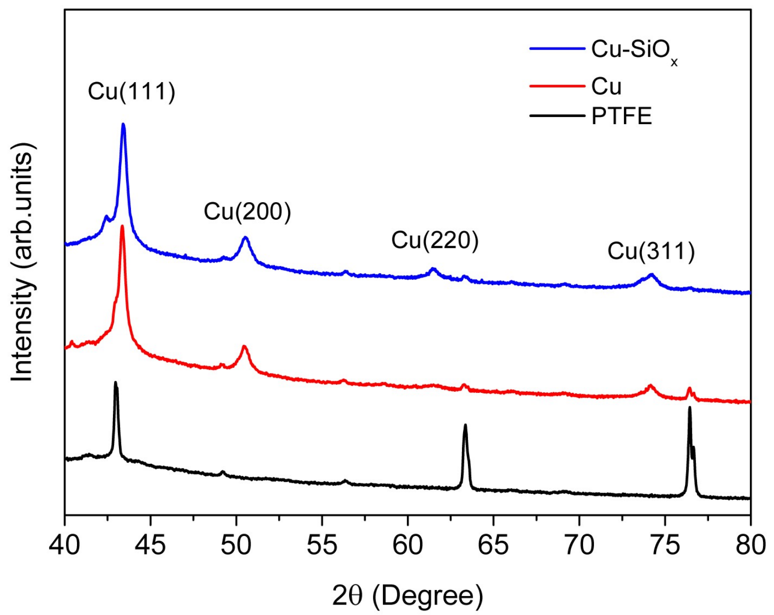

Supplementary Fig. 21 XRD patterns of Polytetrafluoroethylene (PTFE) substrate, Cu and Cu-SiO$_x$.

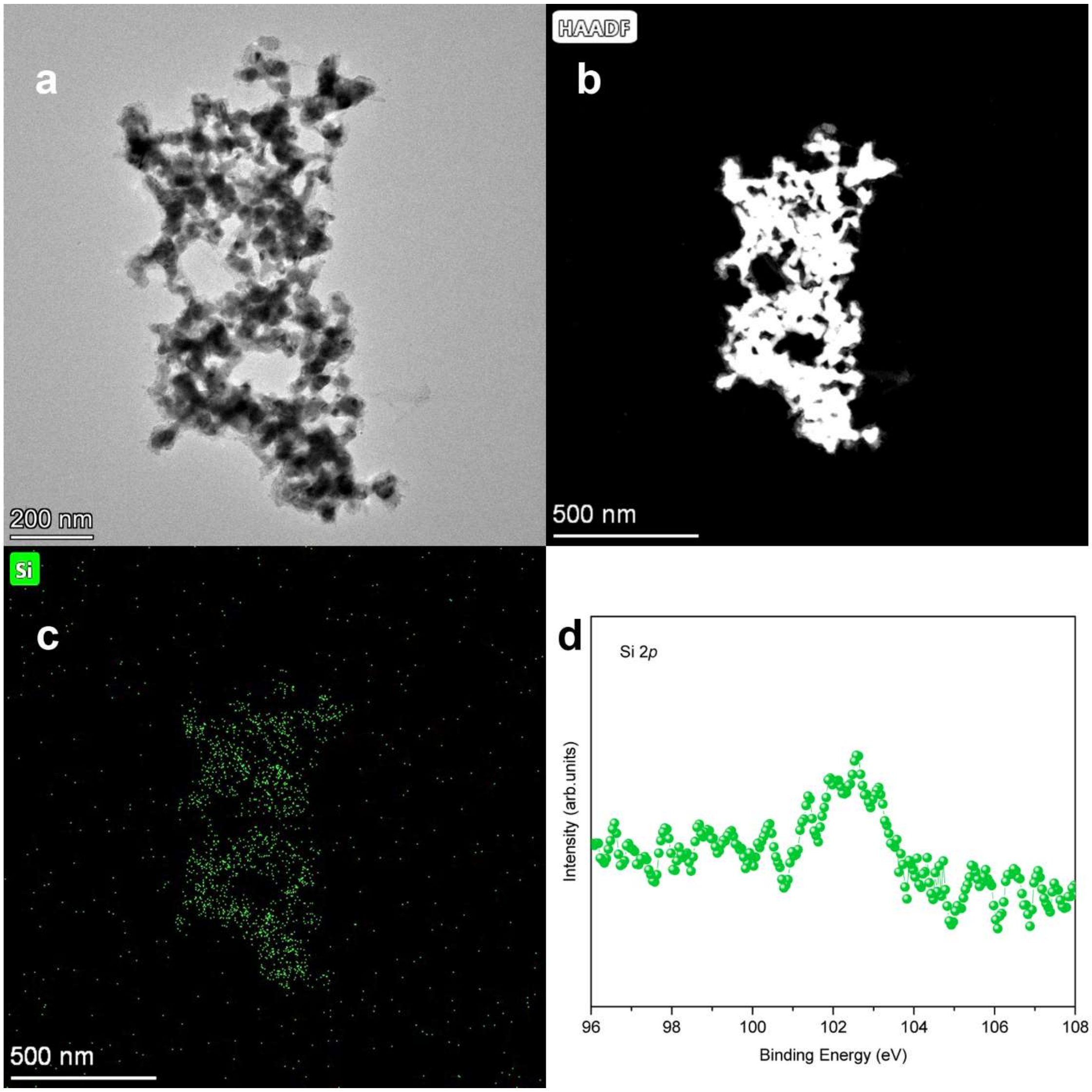

Supplementary Fig. 22 Characterization of 2.5% silica-loaded Cu catalyst before CO_2RR. **a, b** TEM image (**a**) and HAADF-STEM image (**b**). **c** EDX elemental mapping of Si. **d** XPS spectra of Si 2*p*. Results show that Si species are homogeneous distributed at the Cu catalyst and show an oxidation state of +2 (ref.[1]).

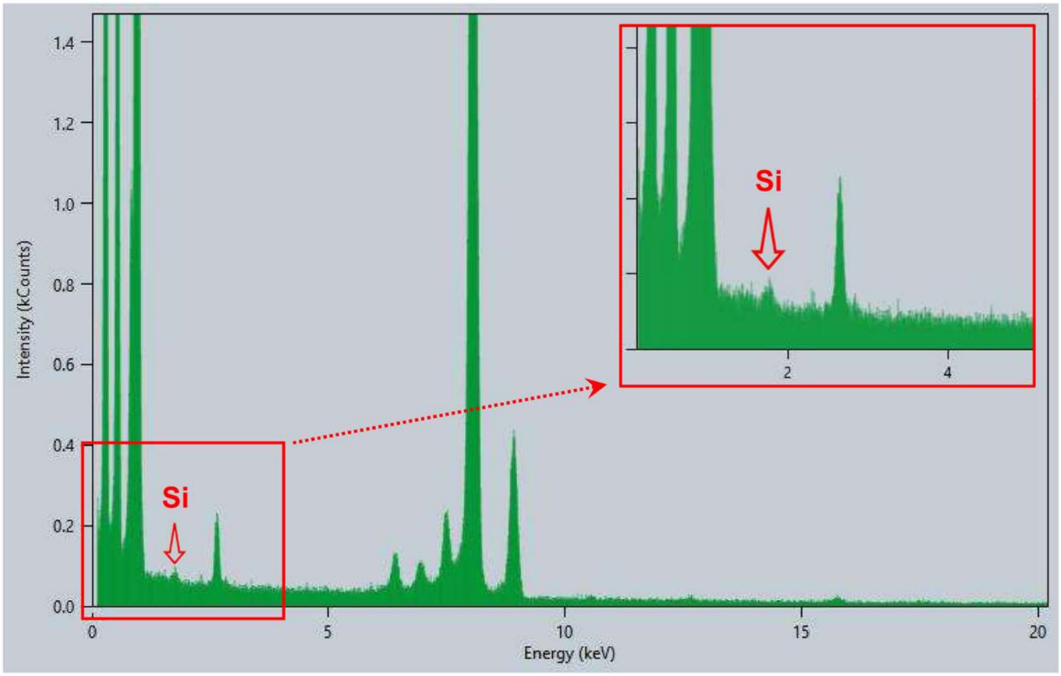

Supplementary Fig. 23 Integrated EDX spectrum of the Cu-SiO_x catalyst after CO_2RR collected from the whole area of **Fig. 2b**.

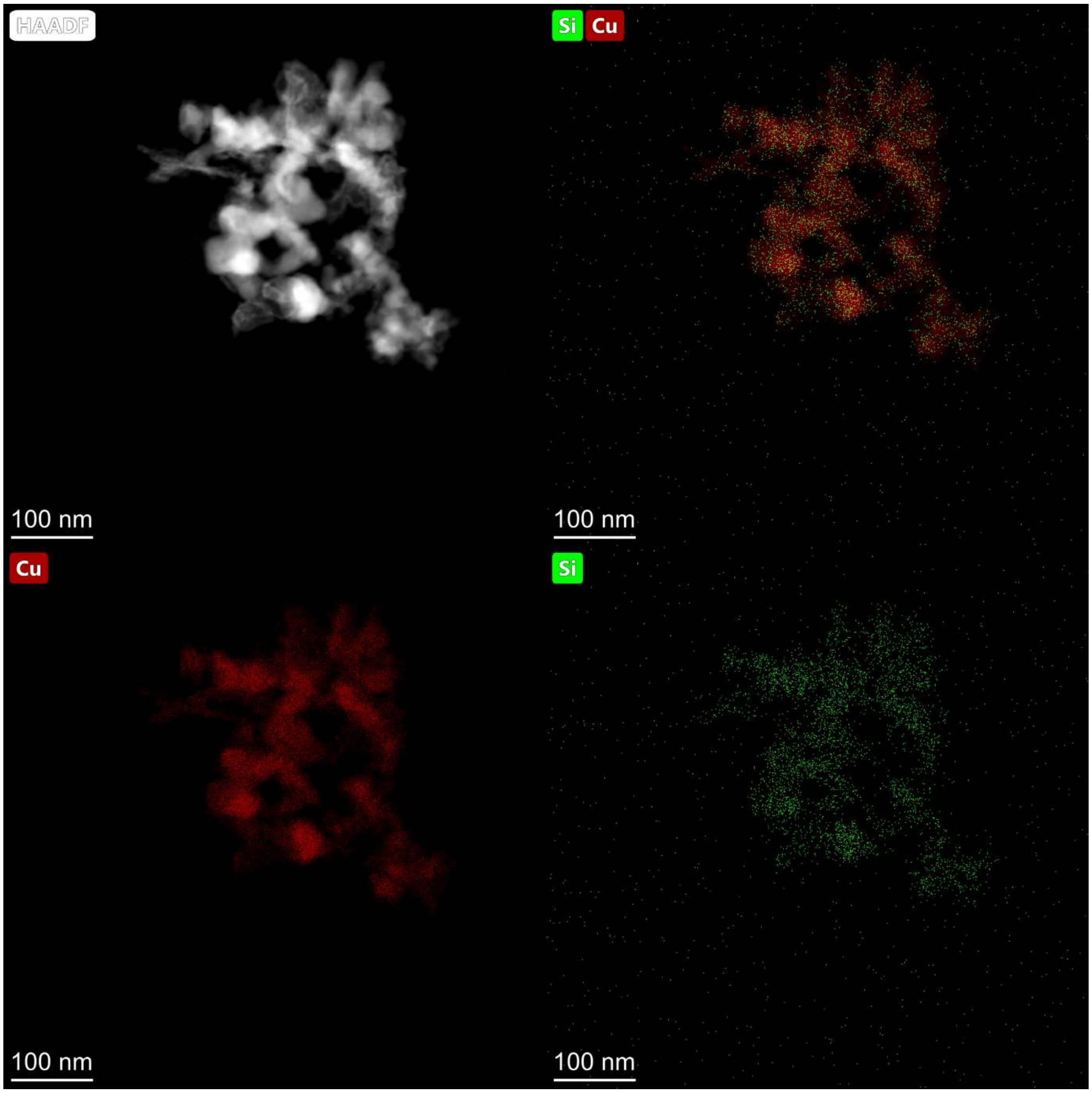

Supplementary Fig. 24 HAADF-STEM image and EDX elemental mapping of 2.5% silica-loaded Cu catalyst after CO_2RR at the region of interest one. Cu is in red and Si is in green.

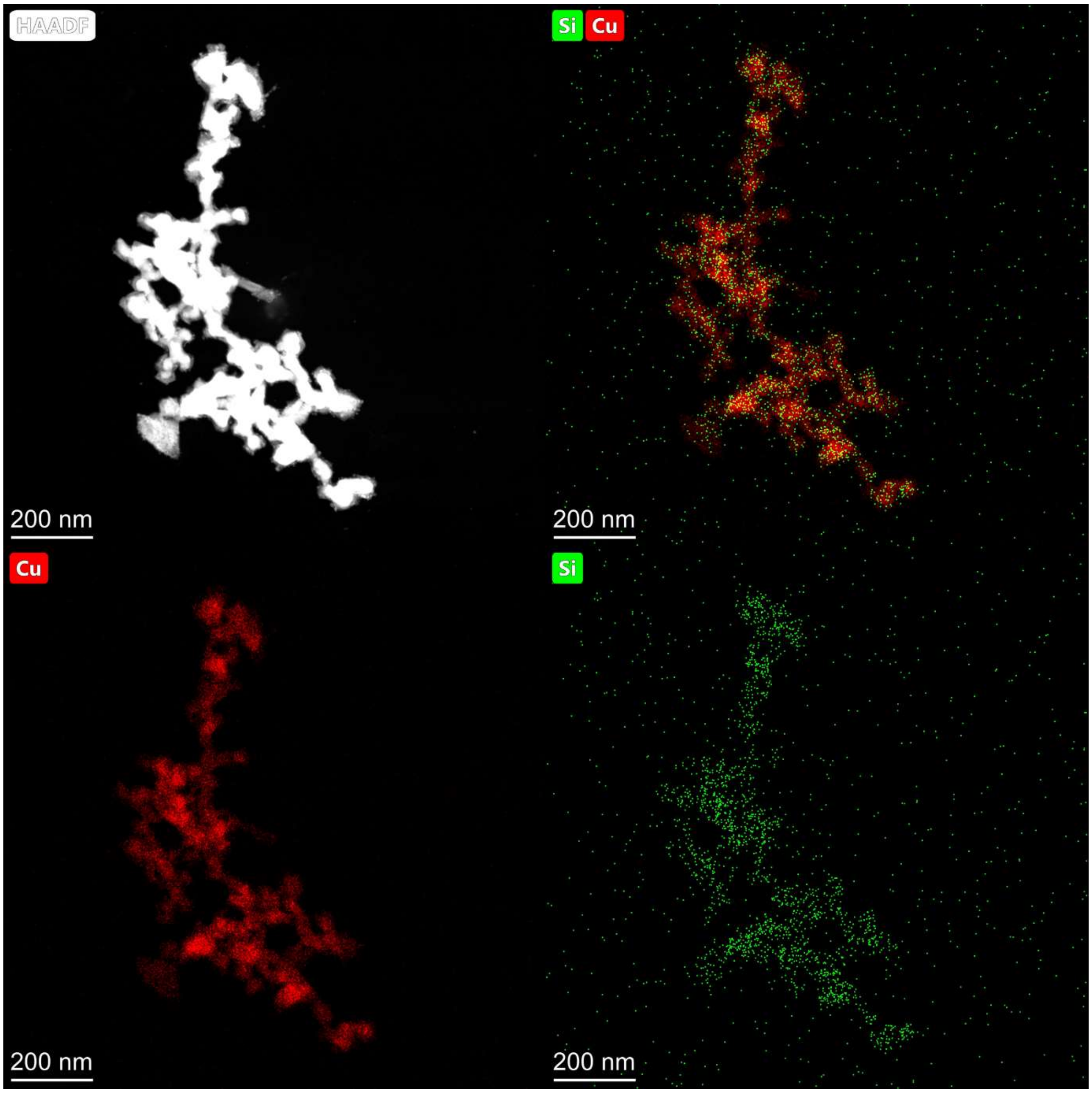

Supplementary Fig. 25 HAADF-STEM image and EDX elemental mapping of 2.5% silica-loaded Cu catalyst after CO_2RR at the region of interest two. Cu is in red and Si is in green.

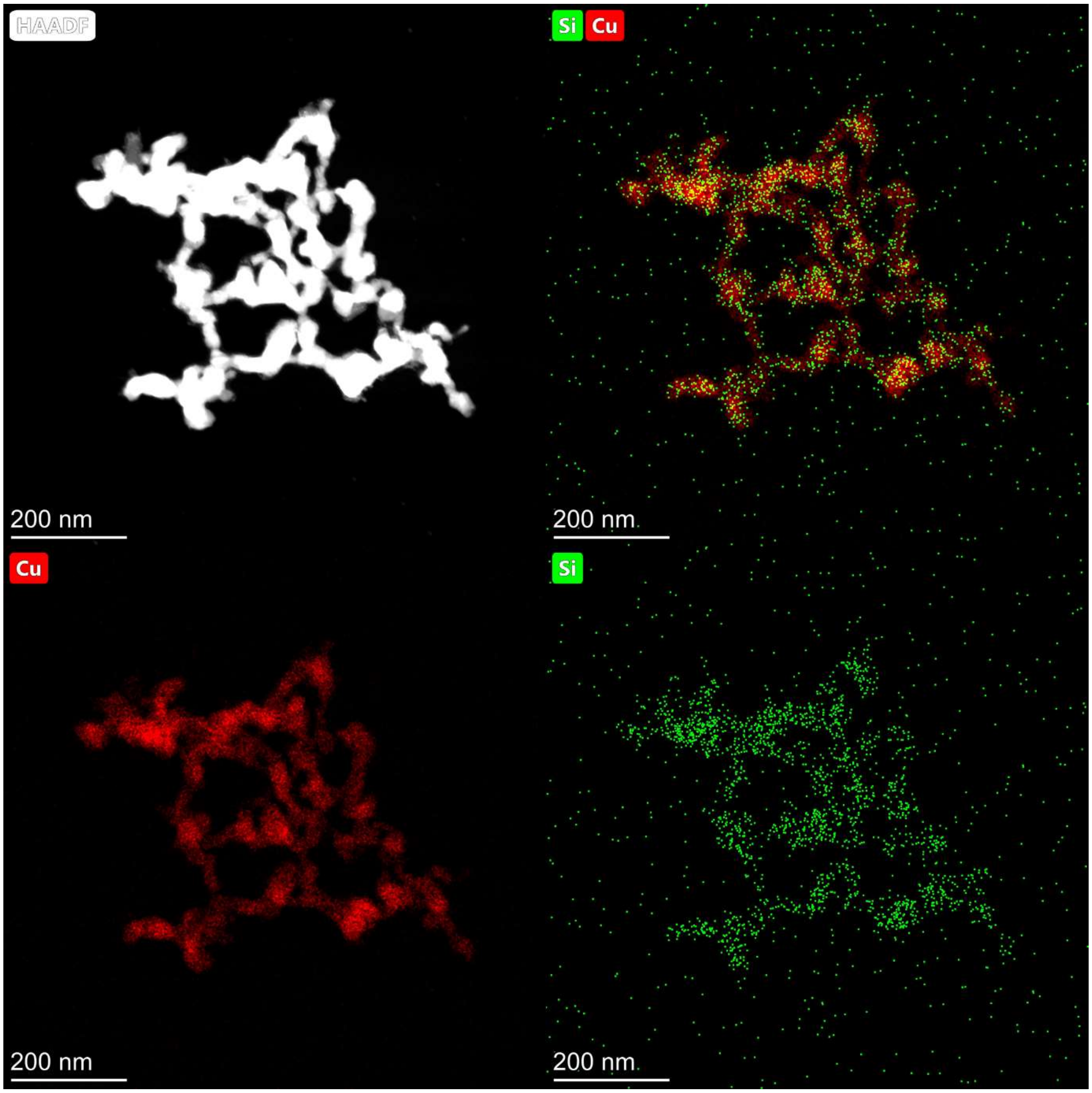

Supplementary Fig. 26 HAADF-STEM image and EDX elemental mapping of 2.5% silica-loaded Cu catalyst after CO_2RR at the region of interest three. Cu is in red and Si is in green.

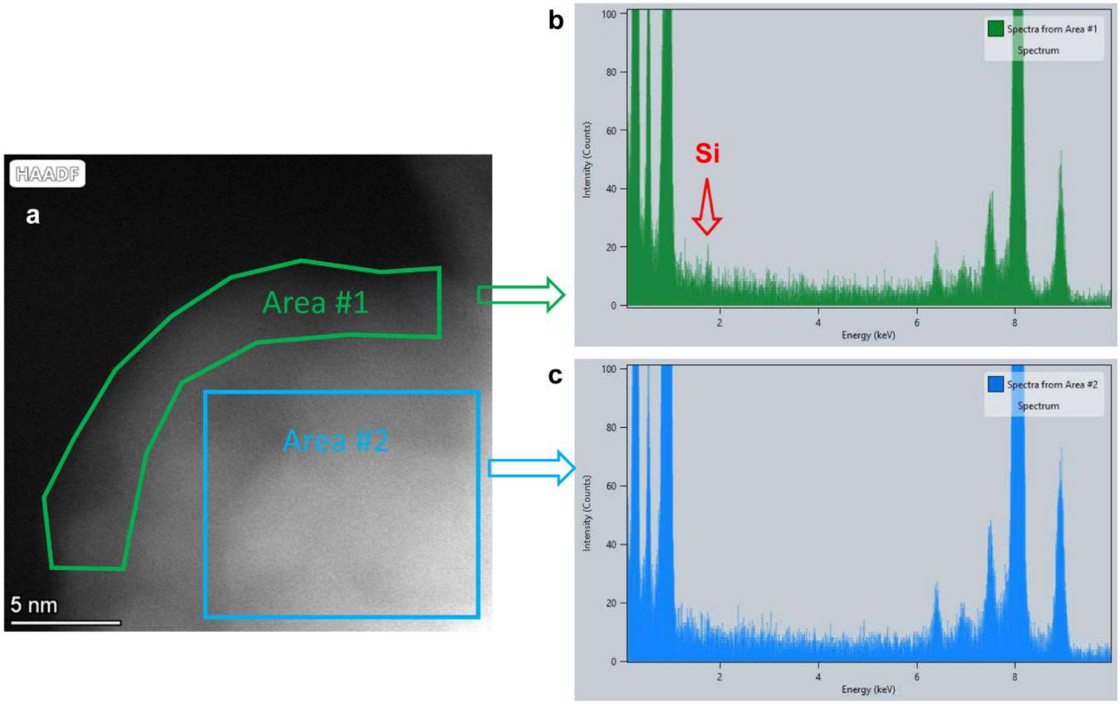

Supplementary Fig. 27 Characterization of 2.5% silica-loaded Cu catalyst after CO_2RR. **a** HAADF-STEM image. **b, c** EDX spectra of the Cu-SiO_x catalyst collected from the surface Area #1 and bulk Area #2 indicated in (**a**), suggesting that Si species are distributed on the Cu surface rather than in the Cu bulk.

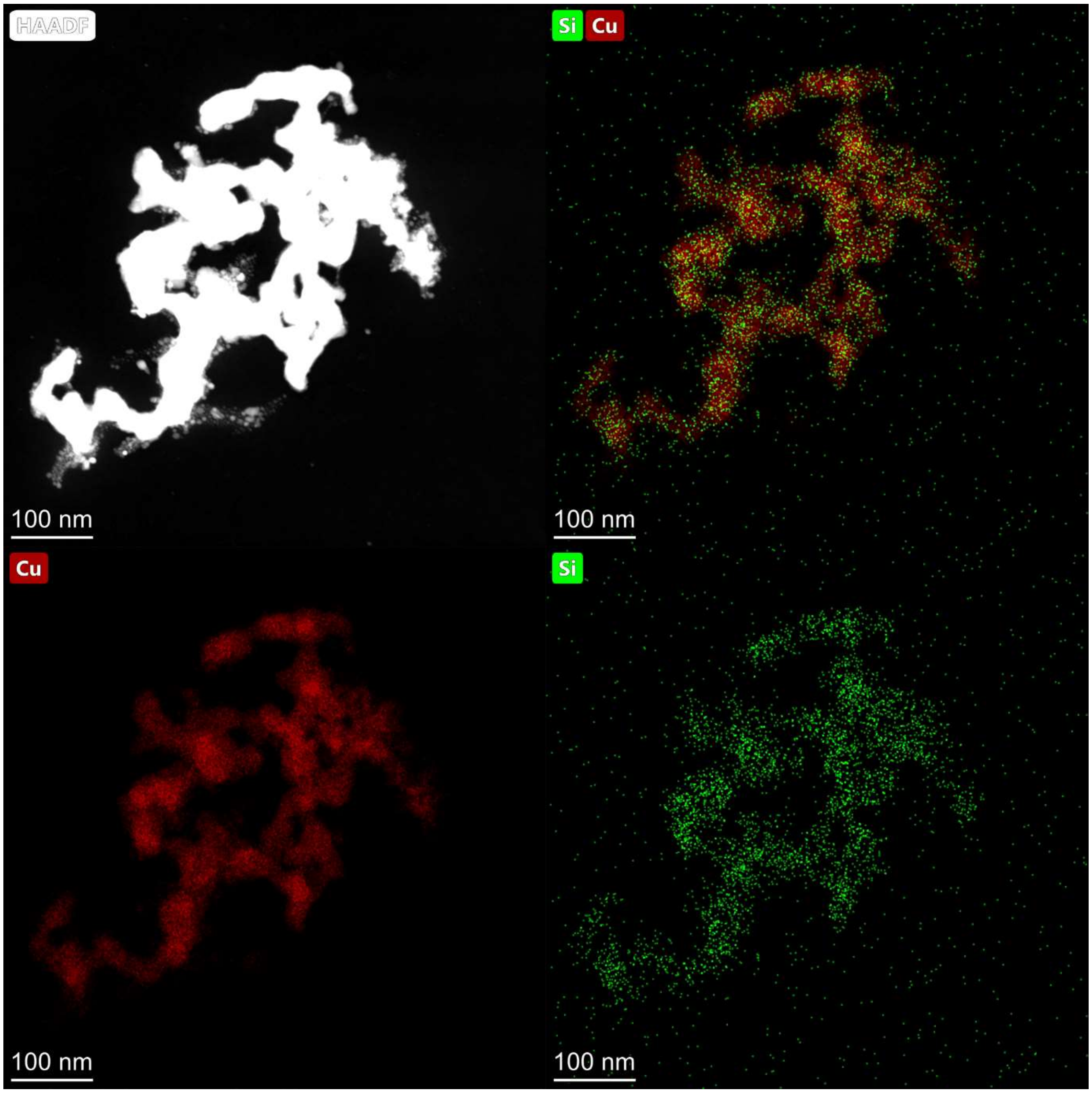

Supplementary Fig. 28 HAADF-STEM image and EDX elemental mapping of 5% silica-loaded Cu catalyst after CO_2RR at the region of interest one. Cu is in red and Si is in green.

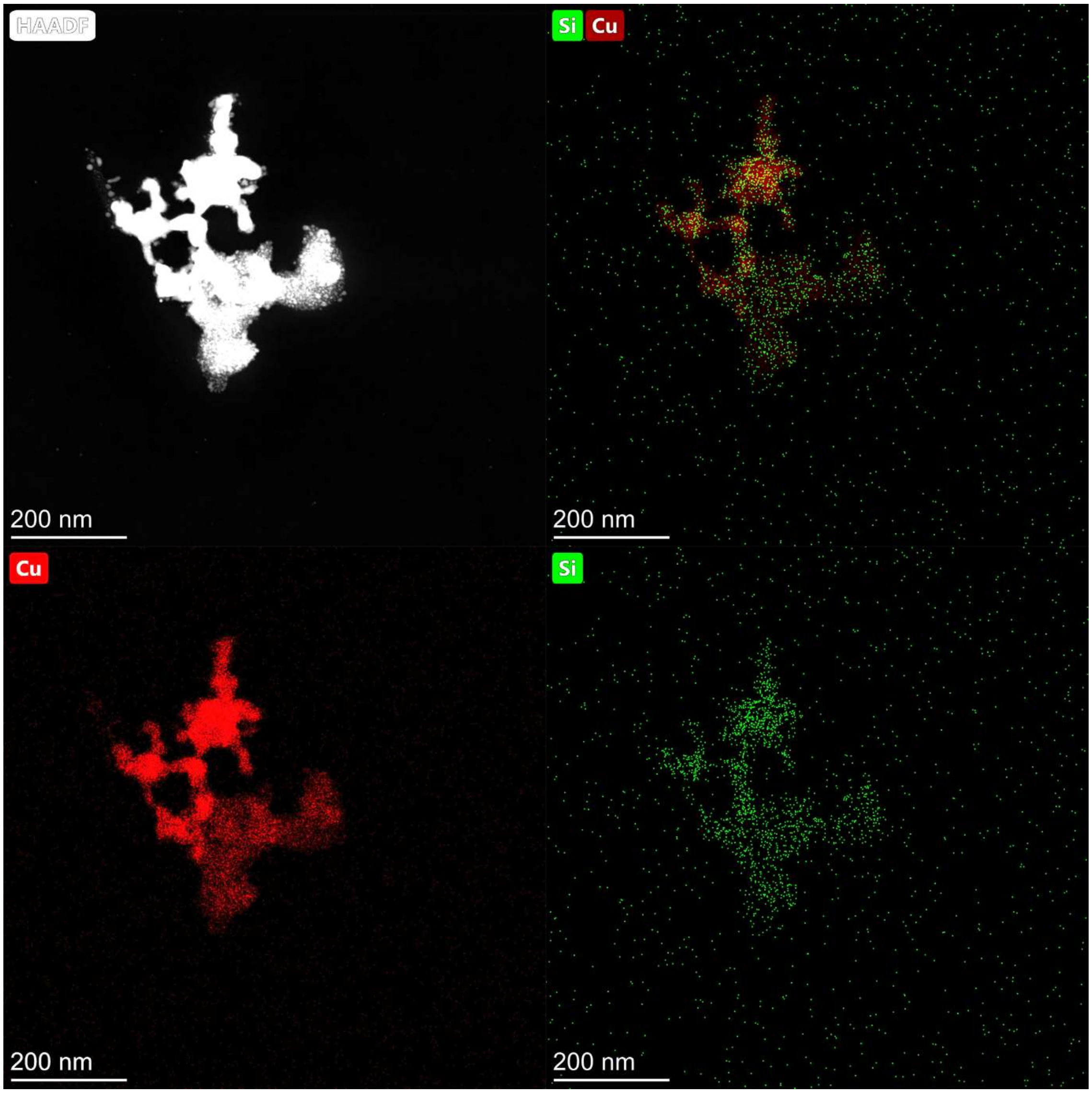

Supplementary Fig. 29 HAADF-STEM image and EDX elemental mapping of 5% silica-loaded Cu catalyst after CO_2RR at the region of interest two. Cu is in red and Si is in green.

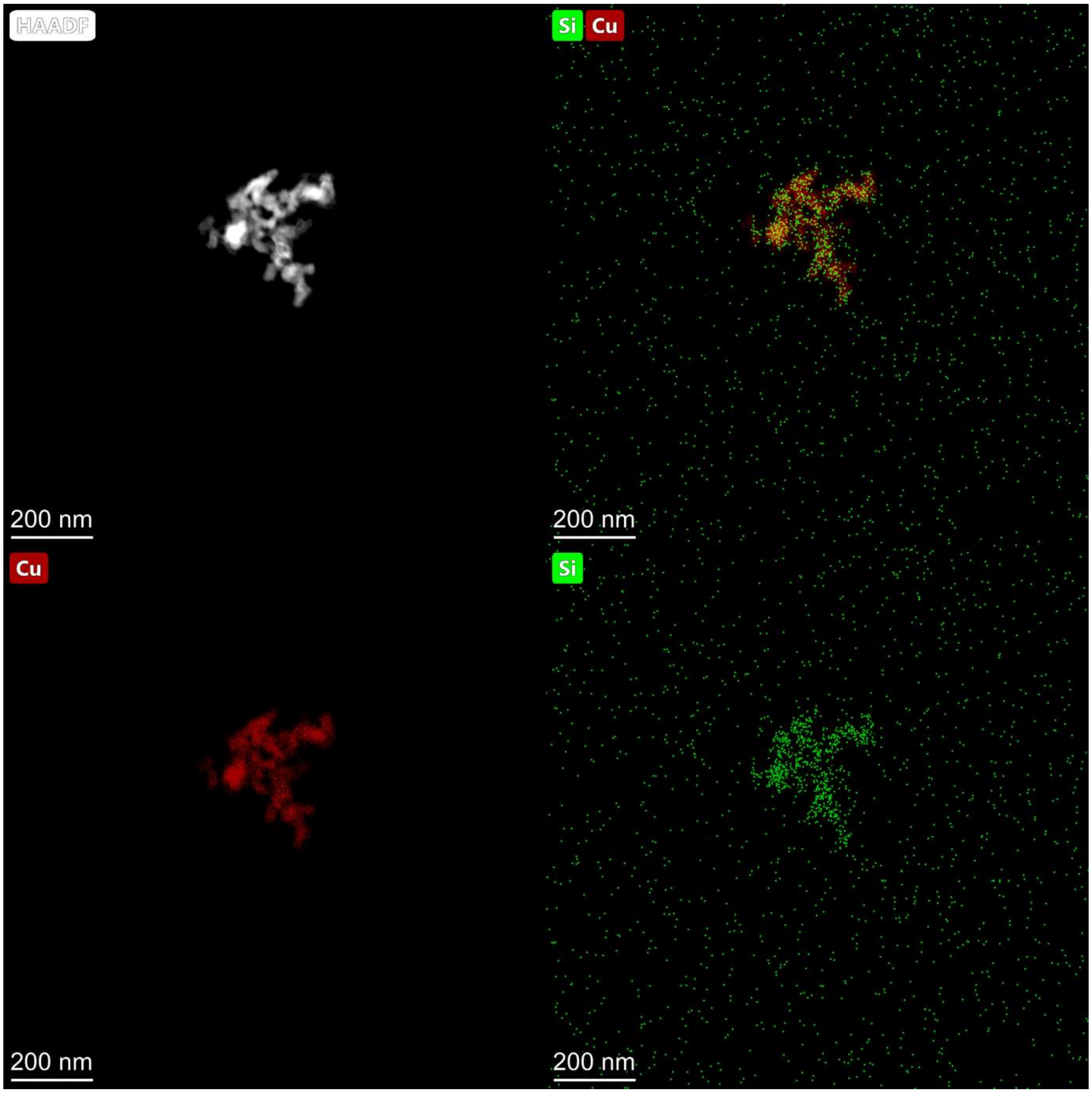

Supplementary Fig. 30 HAADF-STEM image and EDX elemental mapping of 5% silica-loaded Cu catalyst after CO_2RR at the region of interest three. Cu is in red and Si is in green.

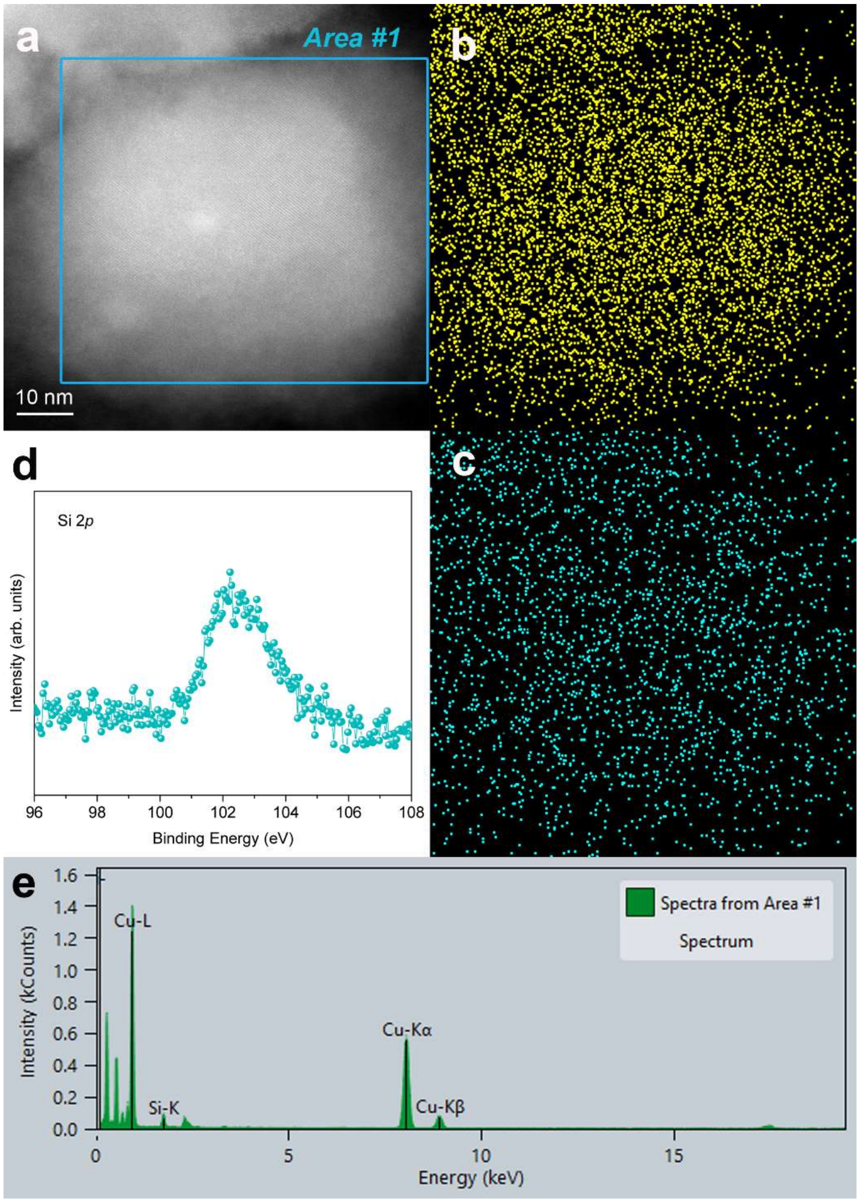

Supplementary Fig. 31 Characterization of 5% silica-loaded Cu catalyst after CO_2RR. a HAADF-STEM image. **b, c** EDX elemental mapping of Cu (**b**) and Si (**c**). **d** The Si 2*p* XPS spectrum. **e** EDX spectrum of the Cu-SiO_x catalyst collected from the Area #1 indicated in (**a**).

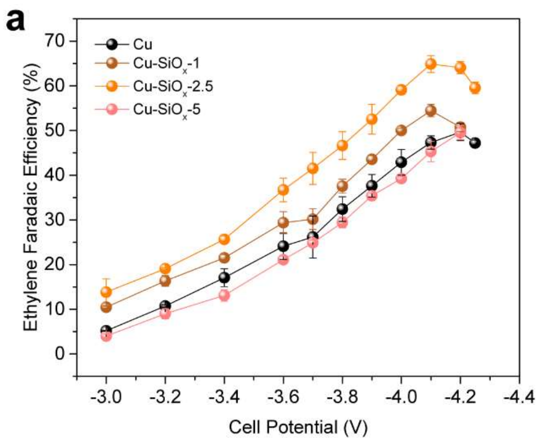

Supplementary Fig. 32 CO_2RR performance of different Cu catalysts loaded in a MEA electrolyzer with 0.1 M $KHCO_3$ anolyte. a, b $FE_{ethylene}$ (**a**) and FE_{CO} (**b**) on different Cu catalysts with a silica loading range of 0% – 5%. Error bars are means ± SD (n = 3 replicates).

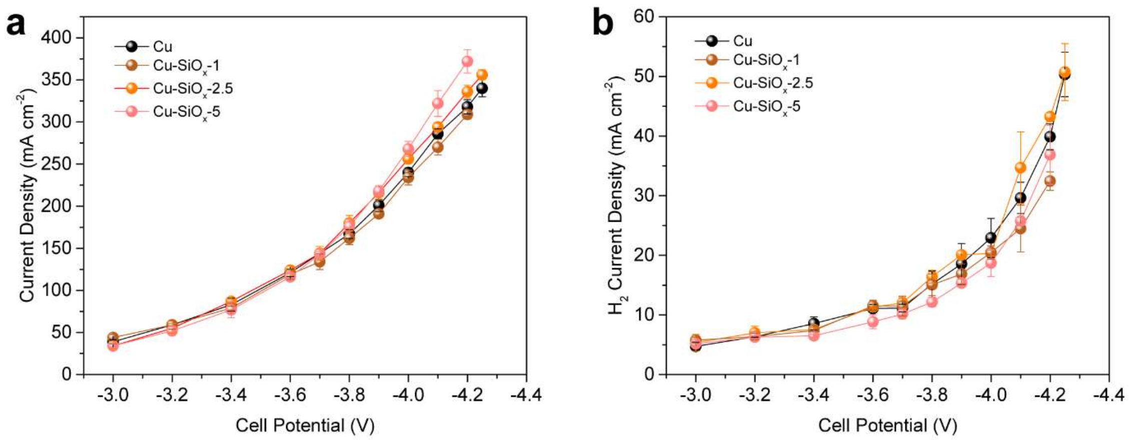

Supplementary Fig. 33 CO_2RR performance of different Cu catalysts in a MEA electrolyzer with 0.1 M $KHCO_3$ anolyte. a, b Total current densities (**a**) and H_2 (**b**) current densities on different Cu catalysts with silica loadings in the range of 0% and 5%. Error bars are means ± SD (n = 3 replicates).

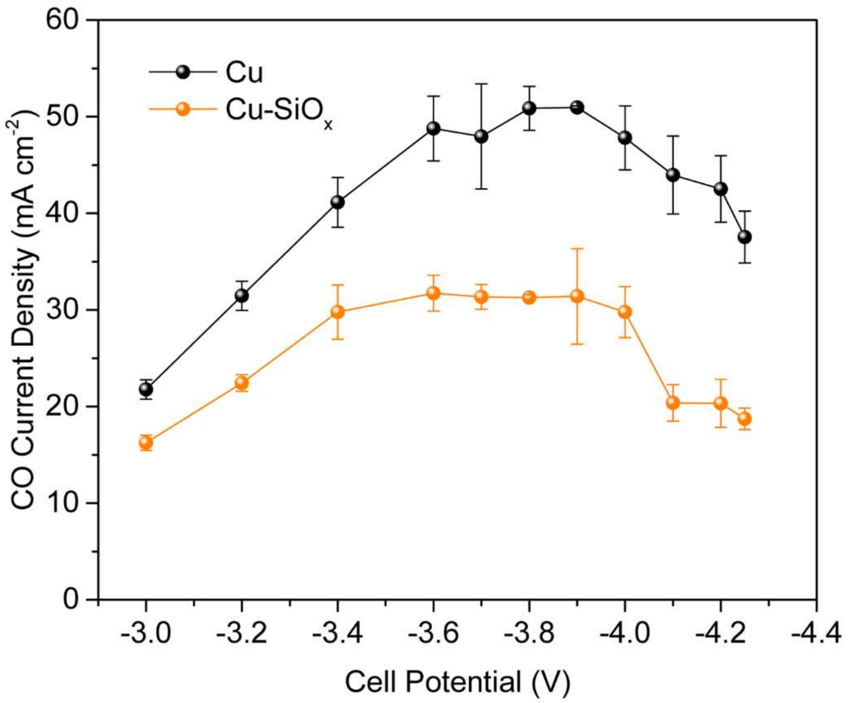

Supplementary Fig. 34 CO current densities on the bare Cu and Cu-SiO_x-2.5 catalysts. Error bars are means ± SD (n = 3 replicates).

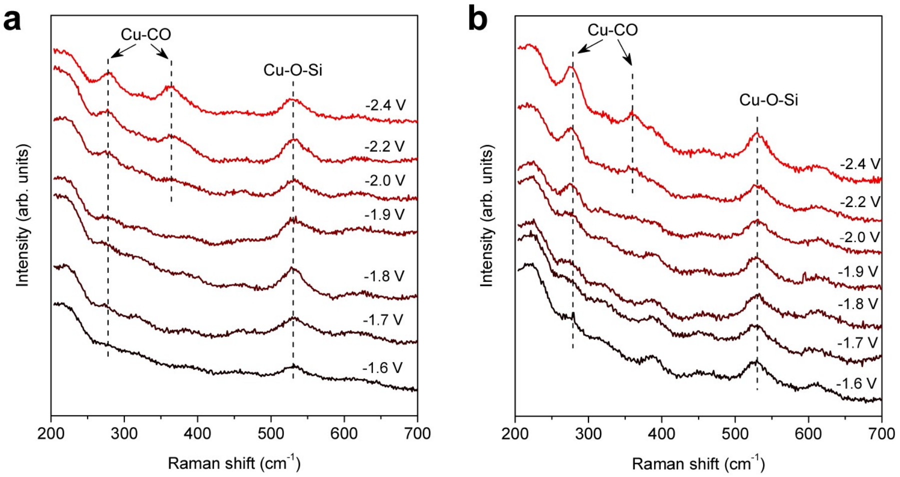

Supplementary Fig. 35 In-situ Raman characterizations. a,b In-situ Raman spectra of Cu-SiO_x-1 (**a**) and Cu-SiO_x-5 (**b**) under different cell potentials. Current density recorded at each condition is listed in **Supplementary Table 4**.

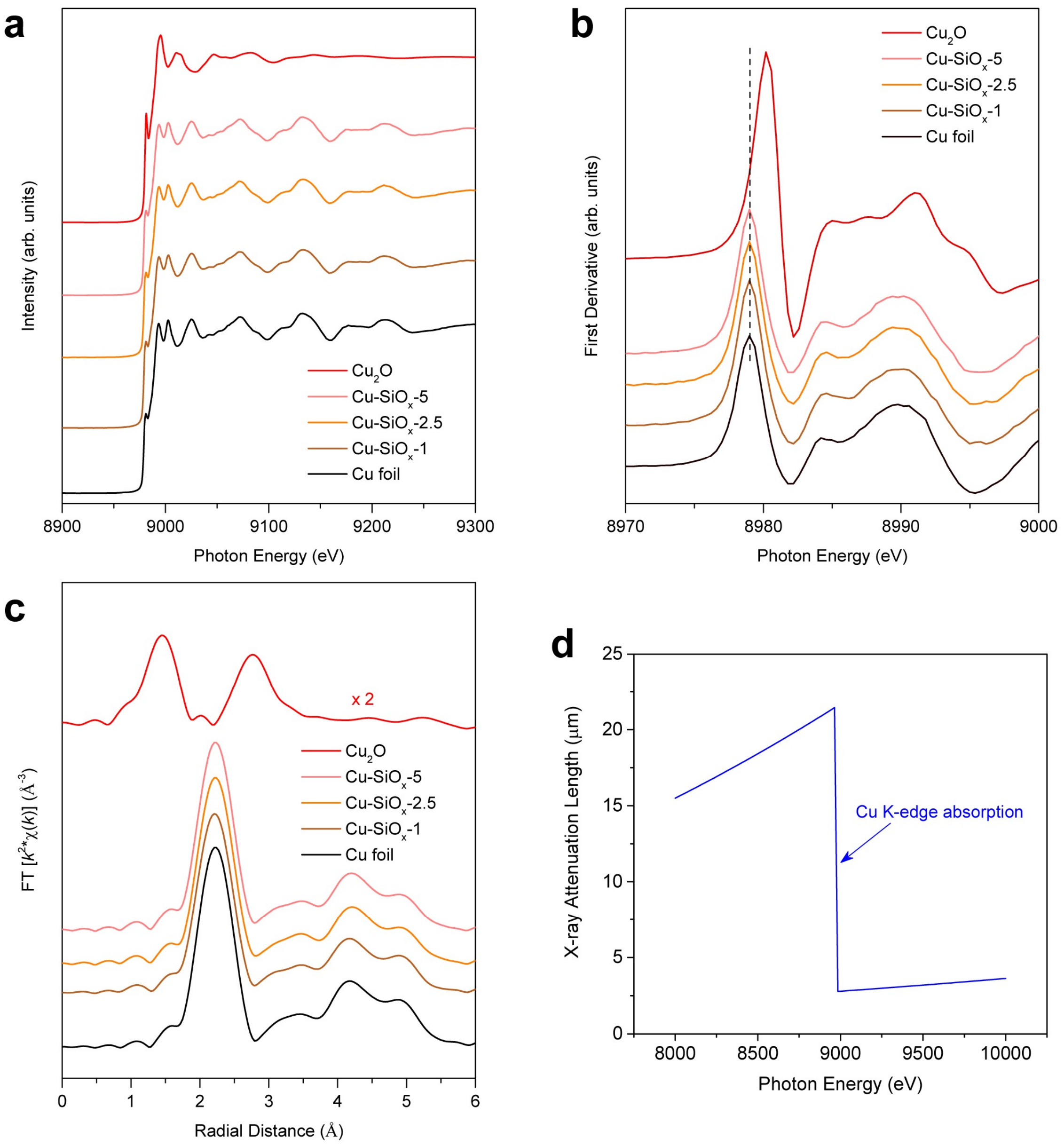

Supplementary Fig. 36 In-situ Cu K-edge XAS. a-c The XANES (**a**), first derivative (**b**) and EXAFS (**c**) spectra of different Cu-SiO_x catalysts and the Cu foil and Cu_2O standards. **d** Calculated X-ray attenuation length vs. photon energy in metallic Cu (density = 8.96 g cm^{-3}). The incident angle is 45 degree (http://henke.lbl.gov/optical_constants/atten2.html).

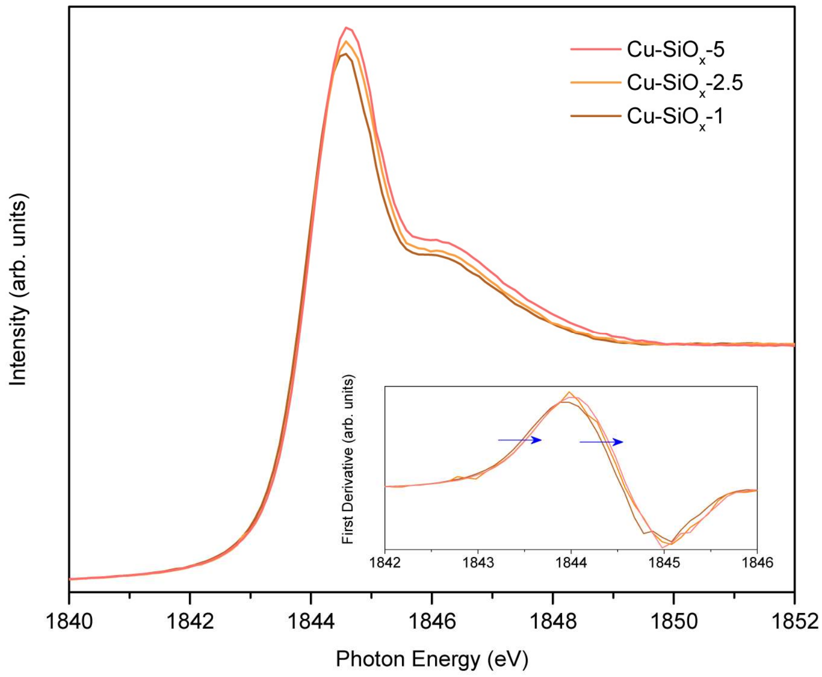

Supplementary Fig. 37 Si K-edge XANES spectra of various Cu-SiO_x catalysts at silica loadings in the range of 1% and 5%. The first derivative spectra is included in the inset to showcase an increase of the Si 1s absorption onset with increasing silica loading.

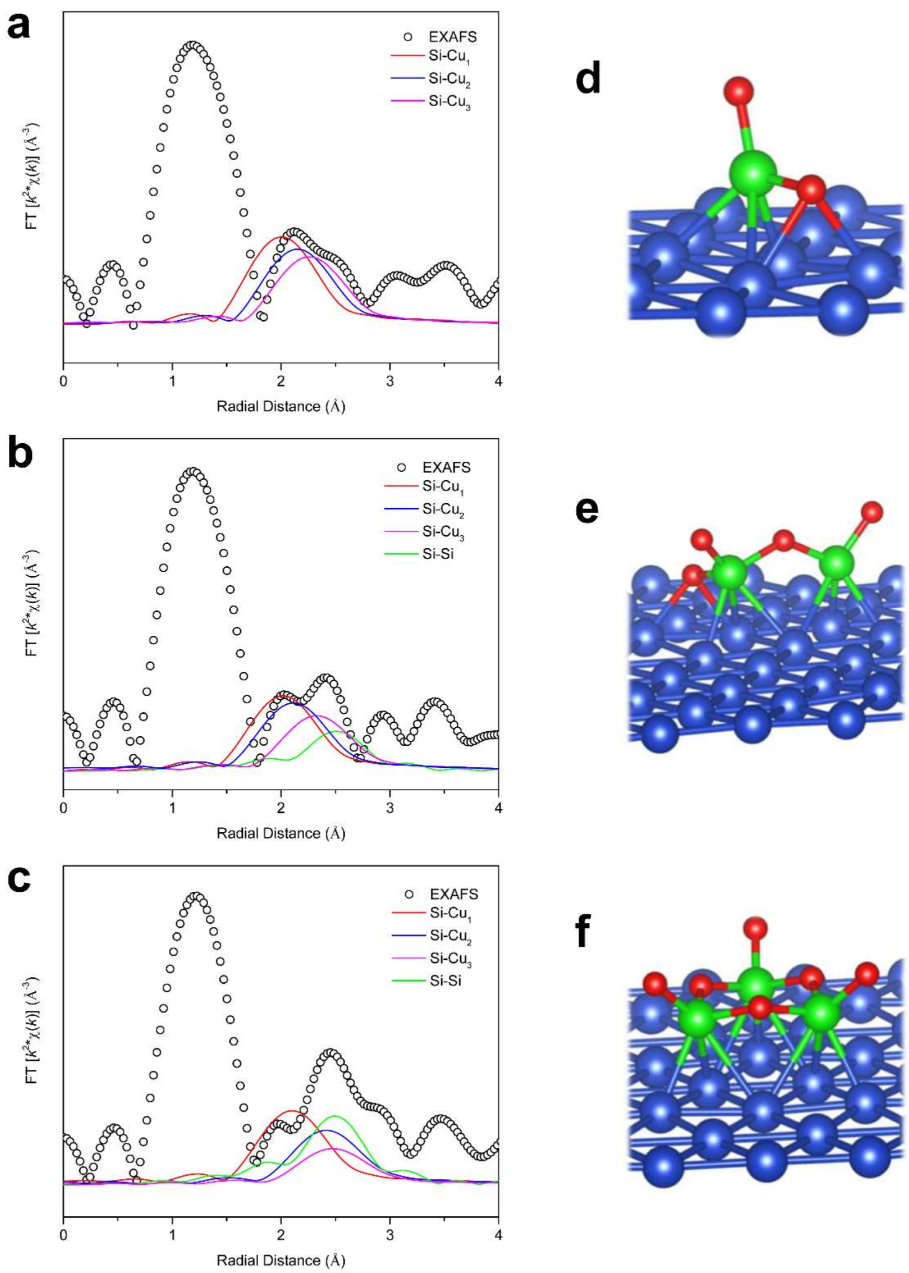

Supplementary Fig. 38 EXAFS fitting of the Si K-edge. a-c Si K-edge EXAFS spectra (open circles) and corresponding scattering paths from EXAFS fitting (solid lines) of various Cu-SiO_x catalysts at a silica loading of 1% (**a**), 2.5% (**b**) and 5% (**c**). **d-f** The relevant material models applied for EXAFS fittings, at a theoretical silica loading of 1.6% in (**d**), 3.1% in (**e**) and 4.7% in (**f**).

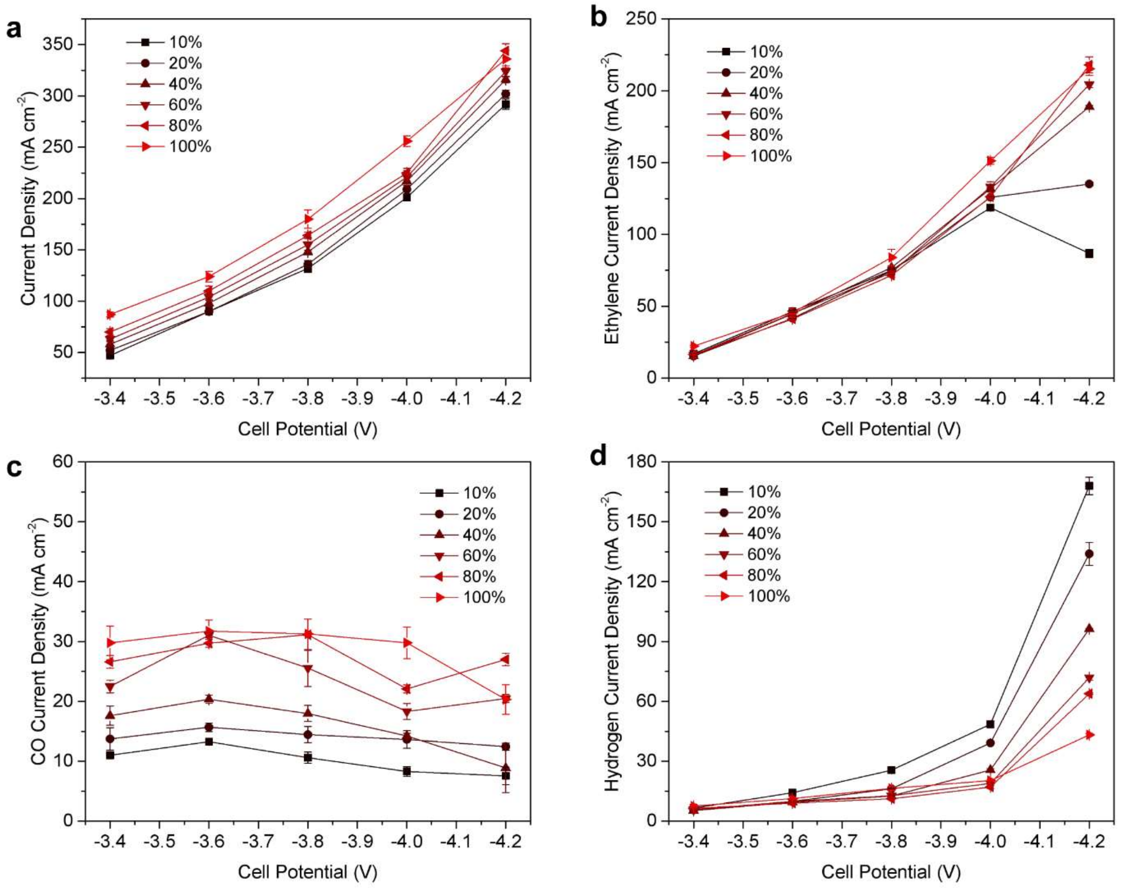

Supplementary Fig. 39 Applied electrochemical performance of 2.5% silica-loaded Cu catalyst integrated into a MEA electrolyzer with 0.1 M $KHCO_3$ anolyte. a-d Effects of CO_2 concentration on total current density (**a**) and partial current densities towards ethylene (**b**), CO (**c**) and hydrogen (**d**). Error bars are means ± SD (n = 3 replicates).

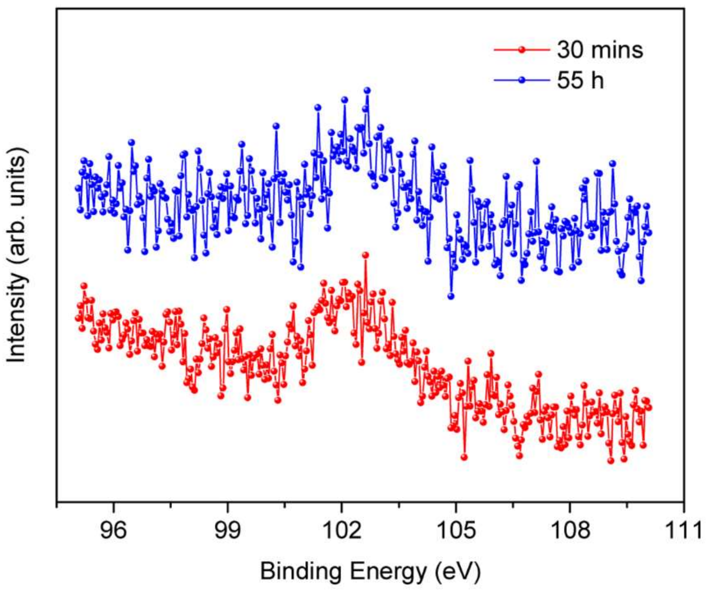

Supplementary Fig. 40 Si 2*p* XPS spectra of the Cu-SiO_x catalyst at a silica loading of 2.5% at different reaction times.

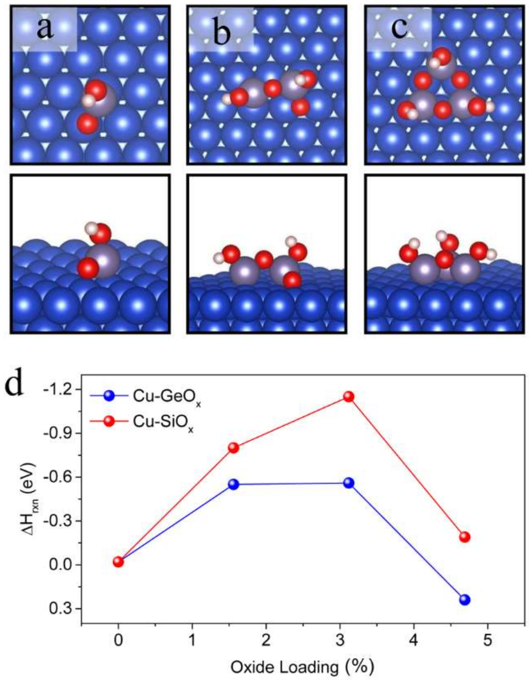

Supplementary Fig. 41 DFT calculations of the Cu-GeO$_x$ catalysts. a-c The energetically favorable Cu-GeO$_x$ catalyst geometries at a GeO$_x$ loading of 1.6% (**a**, 1/16 ML), 3.1% (**b**, 2/16 ML) and 4.7% (**c**, 3/16 ML) over Cu(111). **d** The formation energy (ΔH_{rxn}) of OCCOH* ($CO^* + COH^* \rightarrow OCCOH^*$) over the pure Cu(111) and equilibrium Cu-GeO$_x$ (blue sphere) and Cu-SiO$_x$ (red sphere) catalysts; Volcano-shaped plots are achieved for the formation energies of OCCOH* over the equilibrium Cu-GeO$_x$ and Cu-SiO$_x$ catalyst geometries.

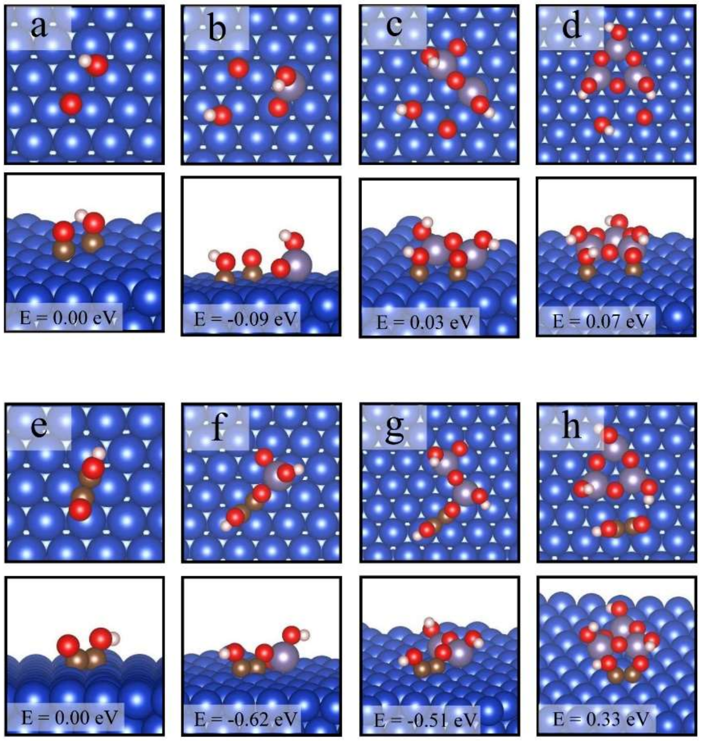

Supplementary Fig. 42 Calculations of the adsorption energies of CO+COH and OCCOH on different Cu catalysts. a-d The energy favourable CO_COH coadsorption over Cu(111) (**a**) and the Cu-GeO_x catalyst at a GeO_x loading of 1.6% (**b**), 3.1% (**c**) and 4.7% (**d**). **e-h** The energy favourable OCCOH adsorption over Cu(111) (**e**) and the Cu-GeO_x catalyst at a GeO_x loading of 1.6% (**f**), 3.1% (**g**) and 4.7% (**h**).

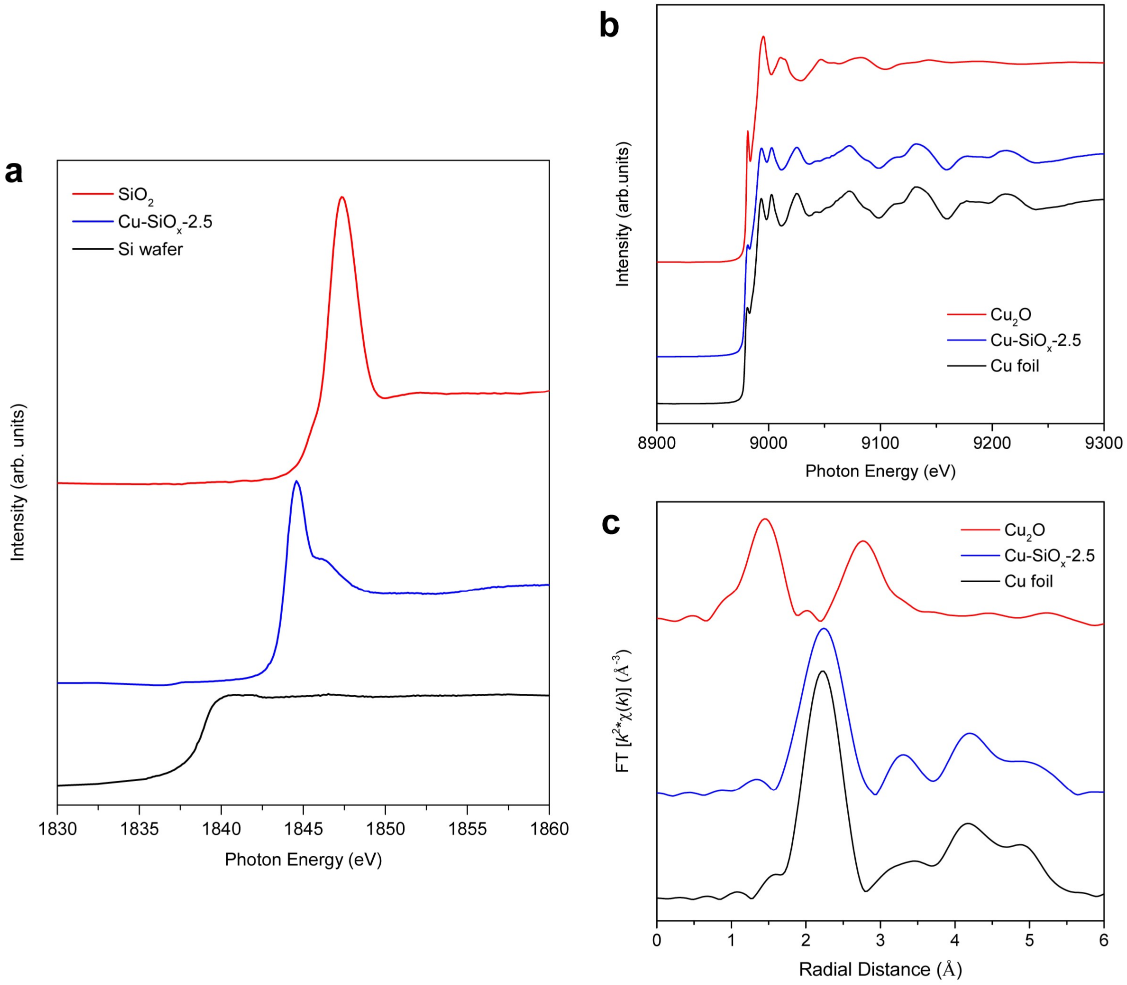

Supplementary Fig. 43 Ex-situ XAS characterization of the Cu-SiO_x catalyst at a silica loading of 2.5%. a The Si K-edge XANES of the Cu-SiO_x catalyst, Si wafer and SiO_2. **b, c** Cu K-edge XANES (**b**) and EXAFS (**c**) of the Cu-SiO_x catalyst, Cu foil and Cu_2O.

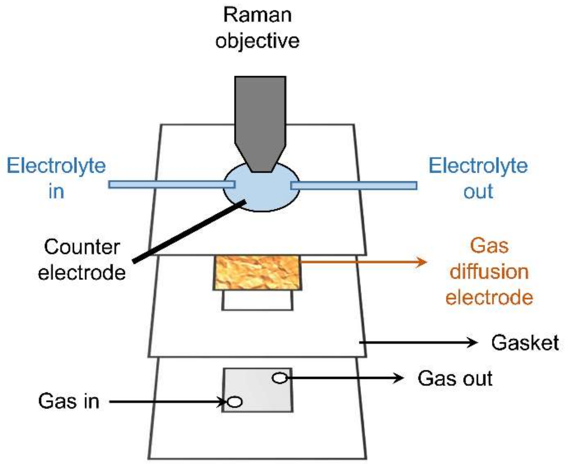

Supplementary Fig. 44 Schematic view of a home-made Raman cell for in-situ analysis.

Supplementary Table 1. The energetics of forming possible geometries of the Cu-SiO_x catalyst at a silica coverage of 1/16 monolayer under hydrogen-proton and water-enriched environments.

Catalyst	Energetics (eV)
Ead_SiO_2	-2.13
SiO_2 +H → SiO(OH)_1	-1.38
SiO(OH)_1→ SiO(OH)_2	1.08
SiO_2 + H_2O → SiO_2_H_2O	-1.16
SiO_2_H_2O → O_$Si(OH)_2$	-0.60
O_$Si(OH)_2$ + H → $Si(OH)_3$	-0.89

Supplementary Table 2. Elemental composition of Si and Cu in different Cu-SiO_x catalysts determined by inductively coupled plasma optical emission spectrometry (ICP-OES).

Sample ID	Si (mg L^{-1})	Cu (mg L^{-1})	Si:Cu ratio
Cu-SiO_x-1	1.57	444.32	0.008:1
Cu-SiO_x-2.5	3.70	398.43	0.021:1
Cu-SiO_x-5	8.01	385.75	0.047:1

Supplementary Table 3. Comparison of electrochemical CO_2-to-ethylene performance for the Cu-SiO_x catalysts vs. the best prior catalysts operating in MEA electrolyzers.

Catalyst	Reaction and cell type	Electro-lyte	$FE_{ethylene}$ (%)	$J_{ethylene}$ (mA cm^{-2})	Reference
**Cu-SiO_x**	**CO_2RR in MEA**	**0.1 M $KHCO_3$**	**65**	**215**	**This work**
Cu/PTFE[2]	CO_2RR in MEA	0.1 M $KHCO_3$	49	100	*Joule* **3**, 2777 (2019)
Molecule/Cu[3]	CO_2RR in MEA	0.1 M $KHCO_3$	64	77	*Nature* **577**, 509 (2020)
Cu:ionomer[4]*	CO_2RR in MEA	0.1 M $KHCO_3$	50	100	*Science* **367**, 661 (2020)

*The electroactive area is 5 cm^{-2} in Figure S33 of Ref[4] for MEA stability testing.

Supplementary Table 4. Current densities recorded at in-situ Raman measurements for different Cu catalysts under various applied cell potentials.

**Cell Potential (V)**	**Cu (mA cm^{-2})**	**Cu-SiO$_x$-2.5 (mA cm^{-2})**	**Cu-SiO$_x$-1 (mA cm^{-2})**	**Cu-SiO$_x$-5 (mA cm^{-2})**
-1.5	4.7	5.2	N/A	N/A
-1.6	6.2	6.8	7.6	7.0
-1.7	8.0	8.9	10.0	9.1
-1.8	10.0	11.3	12.6	11.5
-1.9	12.2	14.1	15.6	14.2
-2.0	14.7	17.1	19.2	17.4
-2.2	N/A	N/A	26.2	23.6
-2.4	N/A	N/A	33.1	30.6

Supplementary Table 5. Comparison of the structures of various Cu-SiO_x catalysts through DFT modeling and fitting of EXAFS experimental data at the Si K-edge. (CN: coordination number, R: bond distance)

Sample	Shell	CN_{DFT}	R_{DFT} (Å)	CN_{EXAFS}	R_{EXAFS} (Å)
Cu-SiO_x-**1**	Si-O	2	1.62	2	1.61
	Si-Cu_1	1	2.30	1	2.26
	Si-Cu_2	1	2.45	1	2.46
	Si-Cu_3	1	2.57	1	2.67
Cu-SiO_x-**2.5**	Si-O	2	1.64	2	1.61
	Si-Cu_1	1	2.32	0.8	2.31
	Si-Cu_2	1	2.42	0.8	2.53
	Si-Cu_3	1	2.63	1.3	2.80
	Si-Si	1	3.0	1	3.04
Cu-SiO_x-**5**	Si-O_1	1	1.64	1	1.74
	Si-O_2	2	1.68	1.7	1.61
	Si-Cu_1	1	2.40	0.8	2.33
	Si-Cu_2	1	2.72	0.7	2.63
	Si-Cu_3	1	2.78	0.7	2.69
	Si-Si	2	2.99	2	2.82

Supplementary Note 1: DFT calculations

For the bare Cu catalyst, we chose Cu(111), which is the most favorable flat surface for the Cu catalyst. A p(4 × 4) supercell and a Monkhorst-Pack mesh[5] of a (3 × 3 × 1) kpoints grid with a plane wave expansion up to 400 eV were chosen for calculating Cu(111). The examined surface had a ~15 Å vacuum layer separation between each periodic unit cell to avoid the lateral interactions from period boundary conditions. To identify the configurations of Cu-SiO_x catalyst at various silica concentrations, we first examined the energetics of different Cu-SiO_x configurations with a low silica surface loading (1/16 monolayer (ML)), as shown in **Supplementary Fig. 2**. When there are solvation and sufficient protons under the CO_2 electroreduction reaction (CO_2RR) conditions, silica can be energetically more favorable to be hydrogenated to SiO(OH) as compared to adsorbed water to form SiO_2-H_2O, $SiO(OH)_2$ and $Si(OH)_3$. In addition, to assess the Si oxidation state, we performed the bader charge analysis for different Cu-SiO_x configurations. Combining the energetics, geometries, and the bader charge analysis, the configuration for the Cu-SiO_x catalyst at a low surface covrage is SiO(OH)_1 with Si oxidation state of +1.87 in **Supplementary Fig. 2**. The energetics of forming possible geometries of the Cu-SiO_x catalyst at a silica coverage of 1/16 ML under hydrogen-proton- and water-enriched environment are given in **Supplementary Table 1**.

The adsorption energy of silica over Cu and the adsorption energy of H_2O over Cu-SiO_x in **Supplementary Fig. 2** were defined as **Supplementary Equation 1-2**, respectively. When hydrogen proton was added to SiO_2, the reaction energy was calculated as **Supplementary Equation 3**.

$$E_{ad_{SiO_2}} = E_{SiO_2\_Cu} - E_{Cu} - E_{SiO_{2gas}} \quad (1)$$

$$E_{ad_{H_2O}} = E_{H_2O\_(SiO_2\_Cu)} - E_{SiO_2\_Cu} - E_{H_2O_{gas}} \quad (2)$$

$$\Delta H_{rxn} = E_{FS} - E_{IS} \quad (3)$$

where $E_{SiO_2\_Cu}$ / $E_{H_2O\_(SiO_2\_Cu)}$ stands for the total energy of the Cu-SiOx catalyst without and with an adsorbed H_2O molecule; and E_{Cu} / $E_{SiO_2\_Cu}$ represents the Cu slab energy or the Cu-SiOx slab energy. $E_{SiO_{2gas}}$ / $E_{H_2O_{gas}}$ stands for the gas phase energy of SiO_2 or H_2O. For **Supplementary Equation 3**, the E_{FS} and E_{IS} represents the total energy of the final and initial states. We employed energy of half of the hydrogen (H_2) in the implicite solvation to represent proton. Negative values of E_{ad} and ΔH_{rxn} indicate that the adsorption of SiO_2 and H_2O or adding proton to SiO_2 is energetically favorable.

We also tested different silica concentrations of Cu-SiOx catalysts, as presented in **Supplementary Fig. 3-4**. The most favourable Cu-SiOx catalyst configurations show that silica is bonded through the Si-O bonds over the Cu(111) surface. The oxidation state of the Si atoms increases with increasing silica concentration. Overall, the average Si oxidation state in the Cu-SiOx catalysts is about +2.

The activation of CO_2 is through proton transfer step, $CO_{2(gas)} + H^+ + e^- \leftrightarrow OCOH^*$. The formation energy (as presented in **Supplementary Fig. 5-7**) was calculated by **Supplementary Equation 4**:

$$E_{form} = E_{(OCOH/slab)} - E_{(slab)} - \frac{1}{2}E_{(H_2(gas))} - E_{(CO_2(gas))} \quad (4)$$

where $E_{(OCOH/slab)}$ and $E_{(slab)}$ represent the total energies for OCOH molecule over a bare Cu or Cu-SiOx slab; E_{slab} is the total energy of a bare Cu or Cu-SiOx slab; $E_{(CO_2(gas))}$ and $E_{(H_2/(gas))}$ are energies of H_2 and CO_2 molecules.

To obtain the most favourable adsorption sites for CO and COH, we examined the adsorption of CO/COH intermediate over different Cu surfaces with and without silica (as shown in **Supplementary Fig. 8-9**). The adsorption energies of CO and COH were calculated by **Supplementary Equation 5-6**:

$$E_{ad} = E_{(CO/slab)} - E_{(slab)} - E_{(CO(gas))} \quad (5)$$

$$E_{ad} = E_{(COH/slab)} - E_{(slab)} - E_{(COH(gas))} \quad (6)$$

where $E_{(CO/slab)}$ and $E_{(COH/slab)}$ represent the total energies for CO and COH molecules, respectively, over a bare Cu or Cu-SiO_x slab; E_{slab} is the total energy of a bare Cu or Cu-SiO_x slab; $E_{(CO(gas))}$ and $E_{(COH(gas))}$ are the gas phase energies of CO and COH molecules, respectively. Here, the more negative value of the adsorption energy represents a stronger binding strength of the CO and COH intermediate at the catalysts surface.

To calculate the reaction energy of the $CO^* + COH^* \rightarrow OCCOH^*$ elementary step over Cu in the presence and absence of silica, we assessed the initial state of the co-adsorption of CO and COH intermediates (**Supplementary Fig. 10-12**) and the final state of the adsorption of OCCOH intermediate (**Supplementary Fig. 13-15**). The co-adsorption energy of CO and COH and the adsorption energy of OCCOH were calculated by **Supplementary Equation 7-8.**

$$E = E_{(CO\_COH/Cu-Si\ \ _x\,slab)} - E_{(Cu-SiO_x\,slab)} - (E_{(CO\_COH/Cu\,slab)} - E_{(Cu\,slab)}) \quad (7)$$

$$E = E_{(OCCOH/Cu-Si\ \ _x\,slab)} - E_{(Cu-Si\ \ _x\,slab)} - (E_{(OCCOH/Cu\,slab)} - E_{(Cu\,slab)}) \quad (8)$$

where $E_{(CO\_COH/Cu-SiO_x\,slab)}$ (or $E_{(CO\_COH/Cu\,slab)}$) and $E_{(OCCOH/Cu-SiO_x\,slab)}$ (or $E_{(OCCOH/Cu\,slab)}$) represent the total energies for CO and COH co-adsorption and OCCOH adsorption over Cu-SiO_x or bare Cu catalyst, respectively; $E_{(Cu-Si\ \ _x\,slab)}$ and $E_{(Cu\,slab)}$ is the total energy of Cu-SiO_x slab or bare Cu slab. Here, the more negative value of the adsorption energy represents a stronger binding strength of the co-adsorbed CO_COH or OCCOH intermediate over the Cu-SiO_x catalyst as compared to that over a bare Cu slab.

The DFT results show that the role of silica in affecting the formation energy of OCCOH from co-adsorbed CO and COH is mainly to lower the adsorption energy of OCCOH via: (1) forming Si-C bond, the C_1 atom of the O=C^1–C^2–OH* intermediate has one electron in a sp^2 orbital that bonds to Cu, while the C^2 atom makes more of a carbene and tends to bond more strongly to Si than Cu, increasing the adsorption energy of OCCOH* and thus decreasing the

formation energy of OCCOH* up to 0.6 eV (**Supplementary Fig. 16-17**); (2) creating a bond between $O^{\delta-}$ of the $OCCOH^*$ intermediate and Si^{2+} in the Cu-SiO_x catalyst, increasing the adsorption energy of OCCOH* and thus lowering the reaction energy of C-C coupling up to ~1.2 eV (**Supplementary Fig. 18-19**). In principle, since Germanium (Ge) is in the same group as silicon, one should expect that C-C coupling can be enhanced by GeO_x modified Cu catalyst. Indeed, based on our DFT calculations, CO and COH co-adsorption remains unchanged and OCCOH adsorption become more stable over GeO_x modified Cu catalyst as compared to the pure Cu catalysts (**Supplementary Fig. 42**). Thus, the reaction energies of C-C coupling over Cu are lowered by the presence of GeO_x. In summary, GeO_x has similar capability as SiO_x to facilitate C-C coupling, although Si exhibites better performance (**Supplementary Fig. 41**).

Supplementary Note 2: Faradaic efficiency and energy efficiency calculations[2]

The FE of each gas product was calculated by **Supplementary Equation 9**:

$$FE_{product} = x_i \times v \times \frac{z_i F P_0}{RT} \times \frac{1}{I_{total}} \times 100\% \quad (9)$$

where x_i represents the volume fraction of gas product i, v represents the gas flow rate at the cathode outlet in s. c. c. m., z_i represents the number of electrons required to produce one molecule of product i, F represents the Faraday Constant, P_0 is 101325 Pa, R represents the ideal gas constant, T represents the temperature, and I_{total} represents the total current.

The FE of each liquid product was calculated by **Supplementary Equation 10**:

$$FE_{product} = n_i \times \frac{z_i F}{Q} \times 100\% \quad (10)$$

where n_i represents the number of moles of product i, and Q represents the charge passed while the liquid products are being collected.

The full-cell energy efficiency (EE) of ethylene was calculated by **Supplementary Equation 11-12**:

$$EE_{ethylene} = \frac{E^o_{cell}}{E_{cell}} \times 100\% \quad (11)$$

$$E^o_{cell} = \frac{\Delta G^o}{-zF} \quad (12)$$

where E^o_{cell} represents the thermodynamic cell potential for ethylene (E^o_{cell} = 1.15 V), ΔG^o represents the change in Gibbs free energy for the reaction, and E_{cell} represents the applied cell voltage (non-iR compensated).

Supplementary References

1 Taylor, J. A., Lancaster, G. M., Ignatiev, A. & Rabalais, J. W. Interactions of ion beams with surfaces. Reactions of nitrogen with silicon and its oxides. *J. Chem. Phys.* **68**, 1776-1784 (1978).

2 Gabardo, C. M. *et al.* Continuous carbon dioxide electroreduction to concentrated multi-carbon products using a membrane electrode assembly. *Joule* **3**, 2777-2791 (2019).

3 Li, F. *et al.* Molecular tuning of CO_2-to-ethylene conversion. *Nature* **577**, 509-513 (2020).

4 de Arquer, F. P. G. *et al.* CO_2 electrolysis to multicarbon products at activities greater than 1 A cm^{-2}. *Science* **367**, 661-666 (2020).

5 Monkhorst, H. J. & Pack, J. D. Special points for Brillouin-zone integrations. *Phys. Rev. B* **13**, 5188-5192 (1976).
